# Heterogeneity of voltage gated sodium current density between neurons decorrelates spiking and suppresses network synchronization in *Scn1b* null mouse models

**DOI:** 10.1038/s41598-023-36036-0

**Published:** 2023-06-01

**Authors:** Jacob M. Hull, Nicholas Denomme, Yukun Yuan, Victoria Booth, Lori L. Isom

**Affiliations:** 1 Neuroscience Graduate Program, University of Michigan, Ann Arbor, MI 48109 USA; 2 Department of Pharmacology, University of Michigan, Ann Arbor, MI 48109 USA; 3 Department of Mathematics, University of Michigan, Ann Arbor, MI 48109 USA; 4 Department of Anesthesiology, University of Michigan, Ann Arbor, MI 48109 USA; 5 Department of Molecular & Integrative Physiology, University of Michigan, Ann Arbor, MI 48109 USA; 6 Department of Neurology, University of Michigan, Ann Arbor, MI 48109 USA; 7 Present Address: Department of Neurology and Neurological Sciences, Stanford University, Stanford, CA 94305 USA

**Keywords:** Neuroscience, Cellular neuroscience, Ion channels in the nervous system, Neural circuits

## Abstract

Voltage gated sodium channels (VGSCs) are required for action potential initiation and propagation in mammalian neurons. As with other ion channel families, VGSC density varies between neurons. Importantly, sodium current (*I*_*Na*_) density variability is reduced in pyramidal neurons of *Scn1b* null mice*. Scn1b* encodes the VGSC β1/ β1B subunits, which regulate channel expression, trafficking, and voltage dependent properties. Here, we investigate how variable *I*_*Na*_ density in cortical layer 6 and subicular pyramidal neurons affects spike patterning and network synchronization. Constitutive or inducible *Scn1b* deletion enhances spike timing correlations between pyramidal neurons in response to fluctuating stimuli and impairs spike-triggered average current pattern diversity while preserving spike reliability. Inhibiting *I*_*Na*_ with a low concentration of tetrodotoxin similarly alters patterning without impairing reliability, with modest effects on firing rate. Computational modeling shows that broad *I*_*Na*_ density ranges confer a similarly broad spectrum of spike patterning in response to fluctuating synaptic conductances. Network coupling of neurons with high *I*_*Na*_ density variability displaces the coupling requirements for synchronization and broadens the dynamic range of activity when varying synaptic strength and network topology. Our results show that *I*_*Na*_ heterogeneity between neurons potently regulates spike pattern diversity and network synchronization, expanding VGSC roles in the nervous system.

## Introduction

Voltage gated sodium channels (VGSCs) are responsible for action potential (AP) initiation and propagation in neurons^1^. VGSCs in brain are composed of one pore-forming α subunit and two non-conducting β subunits (β1 or β3 and β2 or β4), which modulate channel expression, trafficking, and voltage dependent properties^2^. The effects of alterations in VGSC density at the cell surface have been studied extensively, with both increases and decreases being implicated as pathogenic mechanisms in a variety of diseases^3^. Correspondingly, sodium current (*I*_*Na*_) availability is modulated by several neurotransmitter systems and VGSC pore-forming α subunits are the principal targets of a variety of drugs^4,5^. The density of available *I*_*Na*_ is highly relevant for understanding network function both under normal brain states and in pathology. *I*_*Na*_ density impacts neuronal firing threshold and firing rate. These data, when averaged over several neurons, are then used as a measure of the threshold and firing rate of a prototypical neuron. This approach, however, overlooks the role that differences between individual neurons play in shaping the stimulus feature selectivity of individual neurons and in the coordination of network function.

The cell surface density of individual ion channel subtypes varies between neurons^6^. This is the case even between neighboring neurons of the same class and in unambiguously identified same neurons between different animals. Differences in ion channel density can vary over a large range between and within neuronal subtypes^7^. VGSCs are not an exception, with greater than 4-fold ranges reported within populations of wildtype layer 5 and layer 6 cortical pyramidal neurons as well as dopaminergic and GABAergic neurons of the substantia nigra^8–10^. We showed that *Scn1b*^*−*/−^ mice have reduced *I*_*Na*_ heterogeneity between layer 6 (L6) pyramidal neurons, with a 5.3 fold decrease in the coefficient of variation (CV) in current density^9^. Importantly, these data established that *I*_*Na*_ heterogeneity between neurons is subject to disruption by the deletion of a single gene, *Scn1*b. The role of the β1/β1B subunits as regulators of VGSC trafficking, cell surface expression, and transcriptional regulation supports the hypothesis that ion channel heterogeneity between neurons is actively established by specific ion channel regulatory mechanisms rather than by biological imprecision.

What, then is the functional significance of this heterogeneity in *I*_*Na*_ density that normally occurs between individual neurons in control animals? Neuronal heterogeneity impacts neuronal coding by decreasing correlations between neurons and increasing information content within neuronal networks^11,12^. In the neocortex, correlated membrane potential fluctuations within neighboring neurons are high while the correlation of the accompanying neuronal spike times are low, with intrinsic biophysical variability being a potential contributor for active decorrelation^13–16^. Despite the acknowledged role of heterogeneity in neuronal coding and the high degree of neuronal heterogeneity discovered in the brain, we have a limited understanding of the specific mechanisms responsible for enhancing functional heterogeneity between neurons and have only begun to explore how heterogeneity in specific currents such as *I*_*Na*_ impacts network activity.

Here, using genetic, pharmacological, and computational models, we examine the role of *I*_*Na*_ density variability between neurons in patterning of neuronal firing and in network function. We show that *Scn1b*^*−*/−^ cortical L6 and subicular pyramidal neurons, which have reduced heterogeneity of *I*_*Na*_ density, also have more correlated firing between neurons in response to noisy stimuli and impaired heterogeneity in the spike triggered average (STA) current to elicit firing. Pharmacological modulation of *I*_*Na*_ density via administration of a low concentration (3 nM) of the VGSC blocker tetrodotoxin (TTX) similarly alters spike patterns to decorrelate spike trains to levels seen between different neurons, while preserving similar firing rates and spike train reliability. Computational modeling shows that networks of neurons with more variable levels of VGSC conductance (*g*_*Na*_) between neurons generate less correlated spiking, have a higher threshold of coupling strength required for network synchronization, and support a broader dynamic range of network activity when network topology and synaptic strength are varied. Altogether, our results show that *I*_*Na*_ density heterogeneity between neurons is an important mediator of the diversity of neuronal firing patterns and a potent regulator of network function.

## Results

### ***Scn1b*** deletion decreases ***I***_***Na***_ heterogeneity in two distinct pyramidal neuron populations

**Figure 1:**
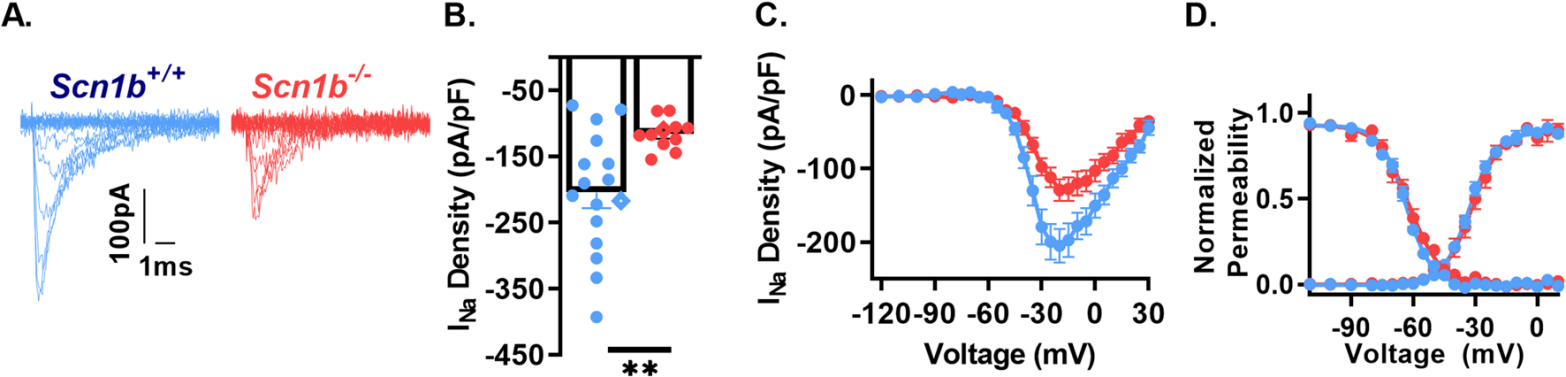
*Scn1b* deletion reduces *I*_*Na*_ density and density diversity in nucleated patches from subicular pyramidal neurons. (**A**) Representative *I*_*Na*_ traces from nucleated patches from subicular pyramidal neurons of *Scn1b*^+/+^ and *Scn1b*^*−*/−^ mice in acute brain slices. Current elicited by depolarizing steps from − 120 mV to + 30 mV from a holding potential of − 120 mV (traces up to peak are shown for visualization of smaller nested currents). (**B**) Peak *I*_*Na*_ density at − 20 mV in nucleated patches as in A n/N = 16/8 *Scn1b*^+*/*+^, 11/8 *Scn1b*^*−*/−^). Diamonds indicate representative traces in A. Asterisks indicate p value (**p < 0.01, Welch’s t-test). (**C**) Current–voltage relationship from recordings in B. (**D**) Normalized voltage dependence of steady state activation and inactivation of recordings in B.

*Scn1b*^*−*/−^ mice have brain region dependent differences in excitability, including altered excitability of pyramidal neurons in cortical L6 and in subiculum. Our previous work showed reduced levels of *I*_*N*a_ heterogeneity between cortical L6 pyramidal neurons, demonstrating that *I*_*Na*_ density variability is subject to dysregulation^9^. Here, we asked whether subicular pyramidal neurons have a similar reduction in *I*_*Na*_ heterogeneity. We used the nucleated patch technique to record from postnatal day (P) 14–21 *Scn1b*^+/+^ and *Scn1b*^*−*/−^ subicular pyramidal neurons. Figure 1A shows representative current traces from *Scn1b*^+/+^ and *Scn1b*^*−*/−^ neurons, with the current–voltage relationship plotted in Fig. 1C. We observed no changes in voltage-dependent properties (Fig. 1D), similar to what we reported previously for L6 *Scn1b*^*−*/−^ neurons^9^. We found reductions in both mean *I*_*Na*_ density (Fig. 1B) (*p* < 0.01, Welch’s t-test) and *I*_*Na*_ density variability between *Scn1b*^*−*/−^ neurons (F = 9.93, *p* < 0.005, Brown-Forsythe test), demonstrating that impaired *I*_*Na*_ density variability in *Scn1b*^*−*/−^ mice is not limited to L6 pyramidal neurons.

### *Scn1b*^*−*/−^ pyramidal neurons have impaired spike train heterogeneity in response to noisy stimuli

**Figure 2:**
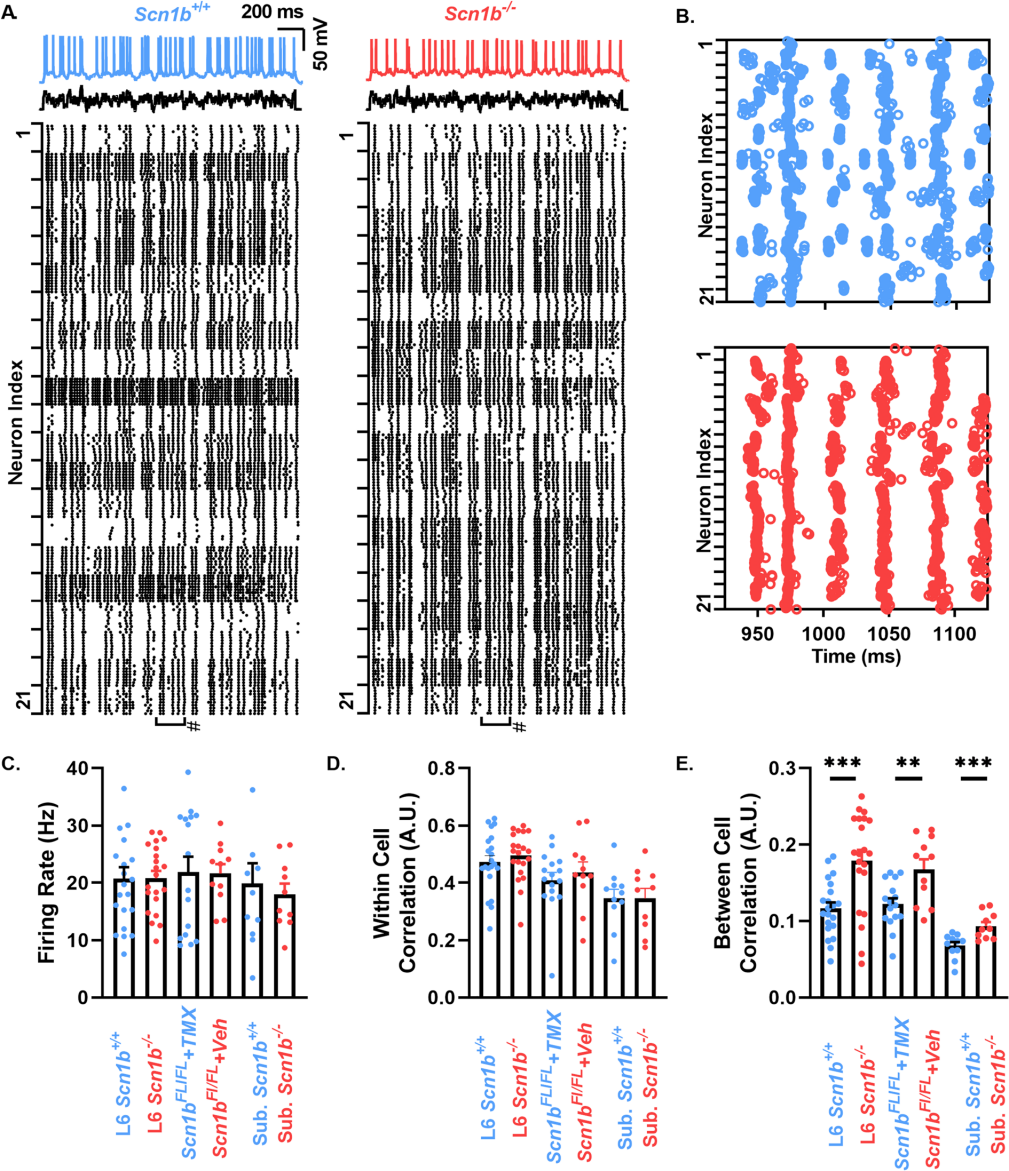
*Scn1b* deletion increases spike train correlation between different neurons. (**A**) (top) Spike trains in a L6 *Scn1b*^+*/*+^ and *Scn1b*^*−*/−^ neuron in response to a time varying stimulus. Raster plots below show the resulting spike pattern of the stimulus above given to 21 cells over ten trials for both *Scn1b*^+/+^ and *Scn1b*^*−*/−^ mice. Boxes marked # indicate the time range expanded in panel B. (**B**) Expanded view of raster plots in A. (**C**) Mean firing rate for L6 *Scn1b*^+/+^ (n/N = 21/6), and *Scn1b*^*−*/−^ (n/N = 21/6) neurons, L6 SlickH/*Scn1b*^Fl/FL^ + Veh (n/N = 16/4) and SlickH/*Scn1b*^FL/FL^ + TMX neurons (n/N = 12/3), and subicular *Scn1b*^+/+^ (n/N = 11/7 *Scn1b*^+/+^) and *Scn1b*^*−*/−^ (n/N = 10/9) neurons. (**D**) Average pairwise correlation between the repeated trials within each cell from cells in C. (**E**) Average pairwise correlation of trains compared between different cells from cells in C. Asterisks indicate p value (**p < 0.01, ***p < 0.001, Welch’s t-test).

Previous work has shown that fluctuating inputs can generate spike trains with high temporal precision over repeated trials in cortical pyramidal neurons, while non-fluctuating inputs generate spike trains with drifting spike times between trials^17^. While this high temporal precision has been shown to hold within the same neuron, an identical fluctuating input generates dissimilar spike trains between neurons of the same class, an effect that was attributed to intrinsic biophysical differences^11^. We thus sought to examine if *Scn1b*^*−*/−^ pyramidal neurons, which have reduced levels of *I*_*Na*_ density heterogeneity compared to *Scn1b*^+/+^ littermates, exhibit impaired spike train heterogeneity in response to a fluctuating stimulus. We recorded from acute brain slices to ask whether spike time correlation or firing rate heterogeneity are impaired in *Scn1b*^*−*/−^ relative to *Scn1b*^+*/*+^ mice in response to fluctuating input. To isolate cell intrinsic differences in firing, fast synaptic transmission was blocked with CNQX (10 µm), APV (50 µm), and bicuculine (10 µm). Fluctuating currents were generated by convolving uncorrelated Gaussian white noise with an alpha function with a τ of 3 ms. This approach was shown previously to be effective in generating reliable spike trains within cortical pyramidal neurons^18^. Figure 2A (top) shows spike trains generated from a mean µ = 100 pA and standard deviation σ = 40 pA current injection in layer 6 pyramidal neurons from *Scn1b*^+/+^ and *Scn1b*^*−*/−^ mice.

Raster plots included below the voltage traces (Fig. 2A, bottom) show the corresponding spike times over ten trials of this stimulus in 21 *Scn1b*^+/+^ and 21 *Scn1b*^*−*/−^ L6 pyramidal neurons, respectively. Figure 2B shows an expanded time view of the raster plots in Fig. 2A at the midpoint of the recording for each genotype. Each of ten trials per neuron are plotted in Fig. 2B, with high clustering within cell spike trains obscuring distinct points at this scale. Total firing rates were not different between genotypes (Fig. 1C). To compare spike correlation between trials of individual cells and between different cells, we converted spike times to vectors of 1 s and 0 s at 1 ms resolution and compared their cross correlation with a time lag of 0. To account for spike time jitter, we set values 1 ms before and after the spike to 1. We then performed pairwise cross-correlation between trials within the same cell (Fig. 2D) and between trials from different cells (Fig. 2E). We averaged values between trials to generate two values per cell, within-cell spike correlation (average of pairwise correlation in trials within the same cell), and between-cell spike correlation (average of pairwise correlation between trials and those of all other cells). The mean correlation within individual neurons was not significantly different between *Scn1b*^*−*/−^ and *Scn1b*^+*/*+^ mice (Fig. 2D), showing that *Scn1b* deletion does not alter the capacity of individual neurons to generate reliable spike trains. Spike trains between cells, however, were more highly correlated in *Scn1b*^*−*/−^ mice compared to *Scn1b*^+/+^ mice (Fig. 2D).

The above recordings were performed in animals with constitutive *Scn1b* deletion. Previous work has shown alterations in neuronal development resulting from *Scn1b* deletion, including deficits in dendritic morphology and neuronal migration that may confound interpretations of the role for VGSC function on spike patterning by introducing structural or neuronal identity defects^2^. To avoid these developmental effects of *Scn1b* deletion, we next performed similar experiments in mice with inducible *Scn1b* deletion. We crossed *Scn1b*^Fl/Fl^ mice with a tamoxifen (TMX) inducible Thy1-Cre (Slick-H) strain, which also expresses a YFP reporter. In this strain, Cre expression is dependent upon TMX administration, thus allowing acute *Scn1b* deletion in pyramidal neurons in adult mice that have undergone normal development, ensuring proper migration and neuronal structure establishment before *Scn1b* deletion. P42-90 *Scn1b*^Fl/FL^ mice were injected with TMX or vehicle and recorded 10–20 days post injection. This protocol was previously shown to induce seizures and eventual lethality in this model^19^. We recorded as described above from YFP-positive (YFP +) L6 pyramidal neurons. Similar to our results in juvenile animals, fluctuating current injection did not result in differences in firing rates between TMX or vehicle treated animals (Fig. 2C), and we observed that *Scn1b* deletion did not impair within cell spike train correlation (Fig. 2D) but enhanced correlation between cells (Fig. 2E).

L6 pyramidal neuron recordings in juvenile mice with constitutive *Scn1b* deletion and in adult mice following induced *Scn1b* deletion resulted in similar enhanced firing correlations between cells. We next asked if subicular pyramidal neurons, which have impaired *I*_*Na*_ density heterogeneity (Fig. 1), showed a similar effect. We found that *Scn1b* deletion had no impact on total firing rate (Fig. 2C) or within cell spike train correlation (Fig. 2D) in subicular pyramidal neurons but did result in increased between cell spike train correlation (Fig. 2E) as described above for L6 pyramidal neurons.

### *Scn1b* deletion impairs spike triggered average heterogeneity

**Figure 3:**
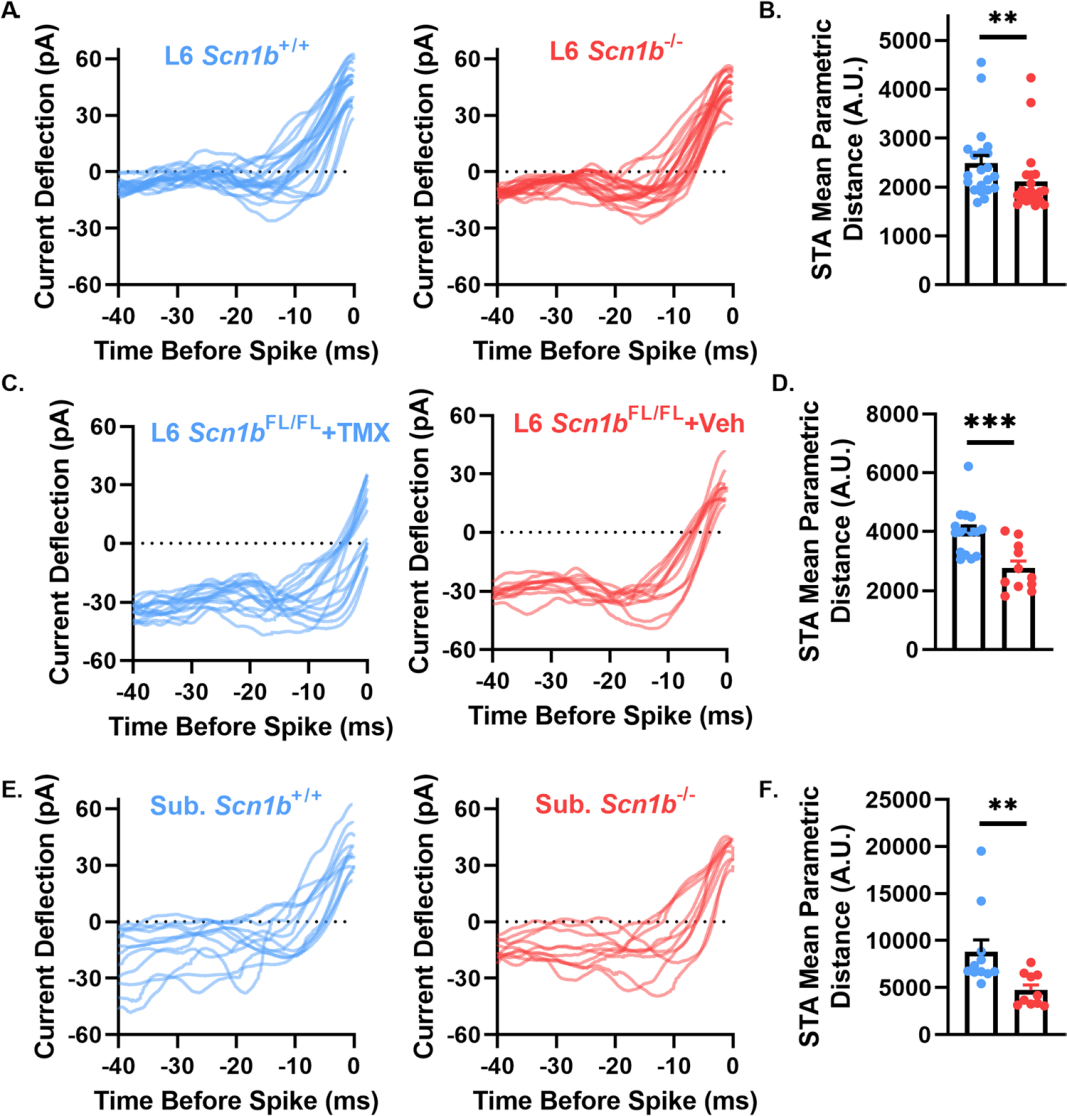
*Scn1b* deletion impairs diversity of the spike triggered average current deflection. (**A**) *Scn1b*^+/+^ (left) and *Scn1b*^*−*/−^ (right) spike triggered averages for each L6 juvenile neuron recorded in Fig. 1. (**B**) Average pairwise STA parametric distance from STAs from other cells as shown in A. **p < 0.01, Welch’s t-test. (**C**) L6 SlickH/*Scn1b*^Fl/FL^ + Veh (left) and L6 SlickH/*Scn1b*^Fl/FL^ + TMX (right) spike triggered averages for each neuron recorded in Fig. 2. (**D**) Average pairwise STA parametric distance from STAs from other cells shown in C. ***p < 0.001. (**E**) *Scn1b*^+/+^ (left) and *Scn1b*^*−*/−^ (right) spike triggered averages for each subicular neuron recorded in Fig. 2. (**F**) Average pairwise STA parametric distance from STAs from other cells shown in E. **p < 0.01.

The spike-triggered average (STA) is a common technique used to measure the preferred stimuli that elicits neuronal firing. Figure 2 demonstrates impaired spike train heterogeneity in response to noisy current injection in *Scn1b*^*−*/−^ pyramidal neurons, suggesting the STA heterogeneity between neurons may also be impaired. Figure 3A, C, and E, show STA traces from individual L6 *Scn1b*^+/+^ and *Scn1b*^*−*/−^ neurons, L6 *Scn1b*^*FL*/FL^ + Veh and *Scn1b*^*FL*/FL^ + TMX, and subicular *Scn1b*^+/+^ and *Scn1b*^*−*/−^ neurons, respectively. To quantify differences in STA variability between conditions, we measured the mean parametric distance between STAs using dynamic time warping (DTW)^20^. Briefly, DTW measures the amount a signal must be deformed to match another signal, providing a convenient distance metric. Here, we show that the mean parametric distance is on average higher in L6 *Scn1b*^+/+^ neurons compared to L6 *Scn1b*^*−*/−^ neurons (Fig. 3B), higher in L6 *Scn1b*^*FL*/FL^ + Veh neurons compared to L6 *Scn1b*^*FL*/FL^ + TMX neurons (Fig. 3D), and higher in subicular *Scn1b*^+/+^ vs. *Scn1b*^*−*/−^ neurons (Fig. 3F). Taken together, these results demonstrate that *Scn1b* deletion impairs the range of stimulus features that elicit firing in multiple populations of pyramidal neurons.

### Altering *I*_*Na*_ density decorrelates spike times between conditions without affecting spike train reliability

We showed in Fig. 1 and in previous work^9^ that L6 cortical pyramidal neurons and subicular neurons have impaired *I*_*Na*_ density heterogeneity. Figure 2 shows that both constitutive *Scn1b* deletion and induced *Scn1b* deletion following normal development lead to increased correlations in neuronal firing. We next asked if moderate *I*_*Na*_ density differences between different neurons could directly contribute to low spike correlation between two otherwise hypothetically identical neurons. We tested this pharmacologically by repeatedly recording a spike train as in Fig. 2 in *Scn1b*^+*/*+^ L6 pyramidal neurons in the absence and presence of 3 nM TTX, thereby modeling two otherwise identical neurons, differing only in a ~ 30% difference in *I*_*Na*_ density. To ensure stability of recording conditions during the time required to record throughout baseline, TTX administration, and washout, we used the perforated patch clamp technique, which allows for preservation of the endogenous intracellular constituents and signaling that can alter cell health over long recordings. We swept continuously every 8 s, giving the same stimulus to compare the effect of 3 nM TTX on spiking patterns.

**Figure 4:**
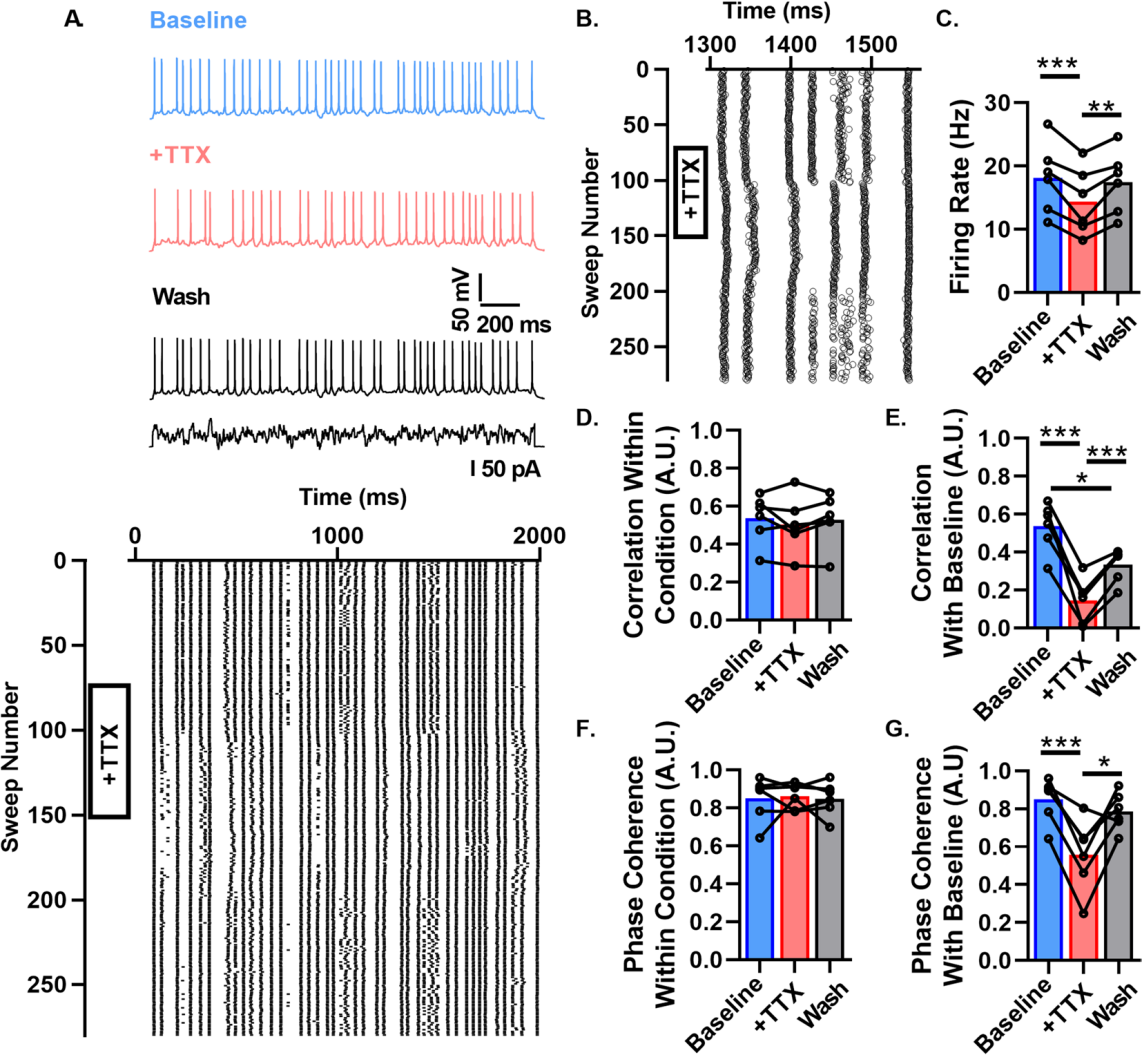
Altering *I*_*Na*_ density decorrelates spiking between conditions without affecting spike train reliability. (**A**) Spike trains in a WT neuron (top), the same neuron in the presence of 3 nM TTX (middle), and washout (bottom) in response to a time varying stimulus. Raster plot below shows the resulting spike pattern of the stimulus above given over repeated trials during addition and wash off of TTX (n = 6, N = 5). (**B**) Expanded view of raster plot in A. (**C**) Average firing rate before, during, and after wash of TTX. (**D**) Average pairwise correlation between the repeated trials within each condition (i.e. Baseline vs. Baseline). (**E**) Average pairwise correlation of trains compared between different conditions (i.e. Baseline vs. + TTX). (**F**) Average phase coherence between the repeated trials within each cell. G. Average phase coherence of trains compared between different conditions. (*p* < 0.05*, *p* < 0.01**, *p* < 0.005***).

Figure 4A shows a representative spike train before the addition of TTX (top), followed by the spike train in the presence of 3 nM TTX (middle), and the spike train after washout (bottom) in a WT neuron. The raster plot at the bottom of Fig. 4A shows each sweep throughout the above recording. Figure 4B presents an expanded time view of these data, showing several representative effects on spike times observed in the recordings, including variable amounts of spike timing shift, missed spikes, novel spikes, unreliable spikes replaced by reliable spikes, and unaffected spikes. We observed a minor decrease in mean firing rate in the presence of TTX relative to baseline and wash conditions (Fig. 4C). TTX did not alter the spike train correlation between trains within the same condition, i.e. comparing + TTX trains vs. + TTX trains shows similar correlation as Baseline vs. Baseline and as Wash vs. Wash (Fig. 4D). These data show that a modest change in *I*_*Na*_ does not reduce the capacity of a neuron to generate reliable spike trains. However, the pairwise correlation between baseline and TTX administration (and TTX vs. wash) conditions was reduced to levels similar to that observed between different neurons shown in Fig. 2 (Fig. 4E). Spike train correlation between the pre TTX condition and the washout condition did not entirely return to baseline levels, likely due to differences in slice/cell health over long recording times (~ 45 min). Because TTX may simply shift all spikes by a similar amount, we used spike phase coherence as an additional measure of spike train similarity. This protocol allowed us to control for a shift in the whole train, as phase coherence would remain unaffected by constant shifts. Phase coherence between trains within conditions was not affected by TTX administration, showing again that altering *I*_*Na*_ density does not impair the capacity of a neuron to generate reliable spike trains (Fig. 4F). However, the phase coherence between trains in the baseline condition vs. those in the presence of TTX was reduced and this effect was removed by washout (Fig. 4G). These results show that heterogeneous *I*_*Na*_ densities between pyramidal neurons can desynchronize the firing of otherwise identical cells in response to fluctuating input without compromising neuronal spike train reliability.

### *g*_*Na*_ density variability between neurons decorrelates spiking in response to fluctuating currents in a computational model across a range of conditions

To systematically investigate the impact of *I*_*Na*_ density variation on firing heterogeneity and spike synchrony between neurons, we examined the effects of variable *g*_*Na*_ on spike patterning in Hodgkin-Huxley style model neurons in response to fluctuating current injections across a range of mean (μ), standard deviation (σ), and time constant (τ). To facilitate comparison with our experimental data, we first generated a model neuron to heuristically recapitulate the single neuron properties we measured previously. We sought to limit model over parameterization with a minimalistic approach, with model neurons limited to 4 ionic currents: *I*_*Na*_ with maximal conductance (*g*_*Na*_)^8^, an A-type potassium current with maximal conductance (*g*_*Ka*_)^21^, a delayed rectifier potassium current with maximal conductance (*g*_*KDR*_)^21^, and a leak conductance^21^. Throughout the modeling, results had cell diameter and *E*_*leak*_ values to approximate the conditions of *Scn1b*^+/+^ L6 pyramidal neurons, as detailed in the Methods section.

**Figure 5:**
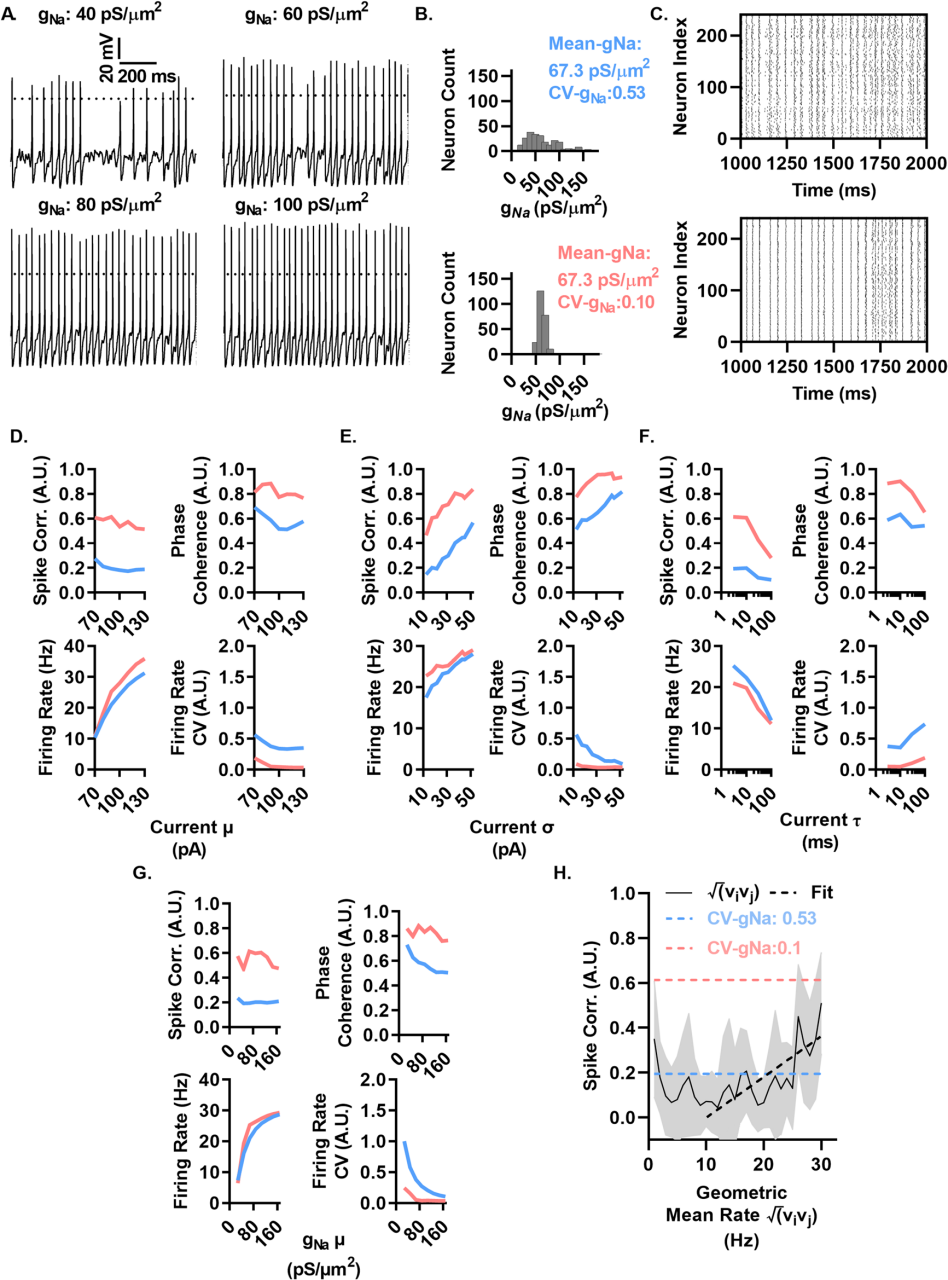
*g*_*Na*_ density variability between neurons decorrelates spiking in response to fluctuating currents in a computational model across a range of conditions. (**A**) Spike patterns of neurons with several levels of maximal sodium conductance (*g*_*Na*_) in response to the same time varying noisy current injection with a mean (μ) of 90 pA, standard deviation (σ) of 22 pA, and time constant (τ) of 3 ms. (**B**) Histograms of the *g*_*Na*_ assigned to neurons used in the simulations in C–H, generated from data in Fig. 1. CV-*g*_*Na*_ denotes the coefficient of variation in *g*_*Na*_ between different cells. Distribution includes 3 values > 180 pS/µm^2^ not shown on the histogram for visibility. (**C**) Raster plots of neuronal spike times of 240 neurons generated from fluctuation currents as in A with *g*_*Na*_ sampled from distributions shown in B. (**D**) Resultant mean pairwise correlation (top left), mean phase coherence (top right), firing rate (bottom left), and coefficient of variation in firing rate (bottom right) between neurons as current μ is varied and σ = 22 pA and τ of 3 ms. (**E**) Results as in D while varying current σ with μ = 90 pA and τ = 3 ms. (**F**) Results as in D while varying current τ and accompanying variation σ which gives a membrane potential standard deviation of 3.7 mV and μ = 90 pA. (**G**) Results as in D while varying g_Na_ μ with current μ = 90 pA, σ = 22 pA, and τ = 3 ms. (**H**) Pairwise correlation between neurons vs their geometric mean rate for 1000 neurons simulated as in C. Black dashed line indicates best fit line between 10 and 30 Hz. Dashed blue line indicates mean correlation over entire CV-*g*_*Na*_ = 0.53 population in C. Dashed red line indicates mean level of correlation over entire CV-*g*_Na_ = 0.1 population in C.

Figure 5A shows representative voltage traces of model neurons with *g*_*Na*_ set to several mean values within the range of *Scn1b*^+/+^ neurons recorded in Fig. 1 and measured previously^9^. These results show the diversity of spiking patterns in response to the same noisy current injection with a mean current (μ) of 90 pA and a standard deviation (σ) of 22 pA, which produces a membrane potential standard deviation of 3.7 mV and a mean voltage of − 53 mV, the mean values measured during whisking behavior in vivo in cortical pyramidal neurons^13^. Figure 5B–G, examines the effect of impairing the coefficient of variation (standard deviation/mean) of *g*_*Na*_ (CV-*g*_*Na*_) as measured in *Scn1b*^+/+^ and *Scn1b*^*−*/−^ neurons previously for L6 pyramidal neurons (0.53 and 0.10, respectively) across a range of conditions^9^. The *g*_*Na*_ in each neuron was determined by sampling a lognormal distribution with a mean of 67.3 pS/µm^2^ with a CV-*g*_*Na*_ of 0.53 (high variance) or 0.10 (low variance). A lognormal distribution was used to circumvent negative and near-zero conductances resulting from sampling a normal distribution. Histograms of *g*_*Na*_ distributions for the above groups of neurons are shown in Fig. 5B.

The high variance *g*_*Na*_ group of neurons generated a highly variable array of spike patterns (Fig. 5C, top), while lowering CV-*g*_*Na*_ to levels observed in *Scn1b*^*−*/−^ neurons resulted in spike trains that were highly stereotyped (Fig. 5C, bottom). To quantify spike train differences resulting from these two distributions, we quantified the spike train correlation, phase coherence, firing rate, and firing rate heterogeneity. The spike train correlation was 0.19 in the high CV-*g*_*Na*_ group of neurons and 0.61 in the low CV-*g*_*Na*_ group. Mean phase coherence was 0.59 in the high CV-*g*_*Na*_ group and 0.88 in the low CV-*g*_*Na*_ group. The mean firing rate was 21 Hz and the firing rate CV was 0.37 in the high CV-*g*_*Na*_ and 25 Hz and 0.05, respectively in the low CV-*g*_*Na*_ group.

To explore the range of input current features over which this effect of *g*_*Na*_ variability influences the resulting spike patterns, we varied parameters of the stimulation current including μ, σ, and τ of the noisy current injections as well as the mean *g*_*Na*_. We found that increasing μ (Fig. 5D) between a range of 70 pA and 130 pA increased the firing rate over a broad range in both groups. Spike correlation and phase coherence remained elevated at all levels of μ measured, indicating the enhanced correlation and phase coherence are not strongly dependent upon mean population firing rate.

Mean population firing rates were also increased by increasing input σ (Fig. 5E), with similar rates in both groups across the measured range. Both spike correlation and phase coherence increased at similar rates in both the high CV-*g*_*Na*_ and low CV-*g*_*Na*_ populations with increasing drive σ. The high CV-*g*_*Na*_ population matched the spike correlation at a σ ~ 5-fold higher than that of the low CV-*g*_*Na*_ population. We next examined the effect of varying τ of the drive current, testing values between 3 and 100 ms. We generated currents with the same μ and σ such that the standard deviation in membrane potential was constant at 3.7 mV across all time constants. We observed that spike correlation and phase coherence decreased in both groups with increasing τ but remained elevated in the low CV-*g*_*Na*_ group at all time constants simulated. We next sought to examine if changes in mean *g*_*Na*_ would influence the effect of CV-*g*_*Na*_ on spike correlation and phase coherence. We simulated networks with mean *g*_*Na*_ ranging from 27.3 pS/μm^2^ to 167.3 pS/μm^2^. We observed that, while the firing rate increases across this broad range, the spike correlation and phase coherence increase resulting from low CV-*g*_*Na*_ persists across this broad range of mean *g*_*Na*_ densities. Previous work has shown that spike correlation increases with increasing rate between pairs of neurons. To examine if this effect could account for or contribute to changes in correlation observed here and in Fig. 4, we simulated 1000 neurons from the high CV-*g*_*Na*_ distribution and examined the relative effect of the increasing geometric mean rate between pairs of neurons. As seen previously, we observe the effect of increasing correlation between pairs of neurons with increasing rate (Fig. 5H)^22^. This effect however fails to account for the majority of differences observed in our simulations, as the relative scale over which this effect manifests and the peak magnitude of this effect is insufficient to account for the differences in correlation alone observed between high CV-*g*_*Na*_ and low CV-*g*_*Na*_ networks shown via dotted lines (Fig. 5H). We fit a line to the dynamic portion of the curve in Fig. 5H and computed the effect that changing the rate by 3.7 Hz, as in Fig. 4, is expected to produce. We found the effect on correlation with a rate reduction of 3.7 Hz is expected to change the correlation by − 0.048, whereas we measured a change in correlation of − 0.39. This result indicates the change in correlation is not accounted for by the phenomenon of increasing correlation with increasing firing rates.

These results show, using a simple computational model, a strong influence of *g*_*Na*_ heterogeneity on spike synchrony and spike patterning under a variety of conditions. These results also establish that *g*_*Na*_ density variability between neurons as recorded previously and in Fig. 1 shifts the necessary magnitude of input fluctuations by a factor of ~ 5 to achieve the same degree of spike correlations observed in homogenous networks, as occurs in *Scn1b* null mice.

### *g*_*Na*_ density variability decorrelates spiking in response to fluctuating synaptic conductances in a computational model

**Figure 6:**
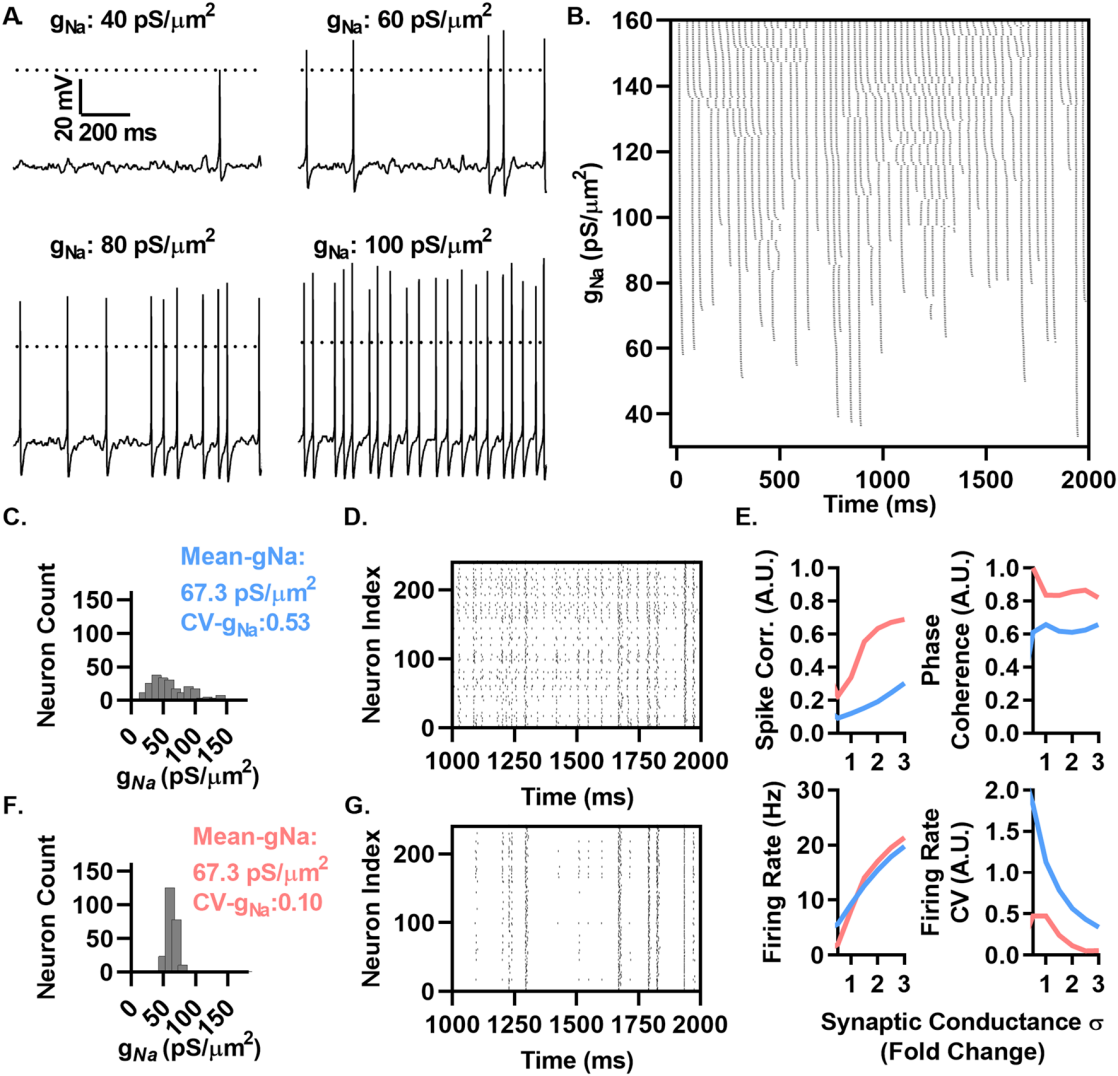
*g*_*Na*_ variability desynchronizes spiking in response to fluctuating synaptic conductances in a computational model. (**A**) Spike patterns of neurons with several levels of maximal sodium conductance (*g*_*Na*_) in response to the same time varying synaptic conductance with µ_e_ = 2.16 nS, µ_i_ = 2.16 nS, σ_e_ = 0.126 nS, and σ_i_ = 0.504 nS. (**B**) Raster plots of 240 neurons to an identical stimulus as in A. where *g*_*Na*_ increases uniformly in neurons between 30 pS/µm^2^ (bottom) to 160 pS/µm^2^ showing successive branching at different levels of *g*_*Na*_. (**C**) and (**F**) Histograms of the *g*_*Na*_ assigned to neurons used in the simulations in D and G, generated from data in Fig. 1. CV-*g*_*Na*_ denotes the coefficient of variation in *g*_*Na*_ between different cells. Distribution includes 3 values > 180 pS/µm^2^ not shown on the histogram for visibility. (**D**) and (**G**) Raster plots of spike patterns of the groups of neurons in C. and F. respectively in response to stimulation as in A. (**E**) The effect of increasing input fluctuations by multiplying the synaptic conductances σ between 0.5 and 3-fold in 0.53 and 0.1 CV-*g*_*Na*_ neurons on firing rate, spike correlation, phase coherence and firing rate heterogeneity.

While noisy current injections can replicate much of the variability in membrane potential in vivo, the synaptic origin of these variations occurs as the consequence of a large number of synaptic events that impact neuronal input resistance and spiking properties. We thus examined firing in response to a fluctuating conductance, which was designed previously to replicate the features of recorded membrane potential fluctuations that occur in vivo^23^. Briefly, the conductance was generated by an Ornstein–Uhlenbeck like process with mean excitatory and inhibitory synaptic conductance, µ_E_ and µ_I_, with standard deviation, σ_E_ and σ_I_. We set these values to achieve a mean membrane potential of − 55 mV and membrane potential standard deviation of 2 mV (µe = 2.16 nS, µi = 2.16 nS, σe = 0.126 nS, and σi = 0.504 nS) and then varied the standard deviation of these synaptic fluctuations between 0.5 and 3 fold. Figure 6A shows representative voltage traces of model neurons with *g*_*Na*_ set to several mean values within the range of *Scn1b*^+/+^ neurons recorded in Fig. 1 and measured previously, showing the diversity of spiking patterns in response to the same synaptic fluctuations. Figure 6B examines the effect of increasing *g*_*Na*_ on the resulting spike pattern to an identical fluctuating synaptic conductance, showing a high dynamic range of altering *g*_*Na*_ to alter spiking patterns over a broad range of *g*_*Na*_ conductance densities. Figure 6C–E examine the effect of impairing CV-*g*_*Na*_ as performed in Fig. 5.

The high variance *g*_*Na*_ group of neurons (Fig. 6C) generated a highly variable array of spike patterns (Fig. 6D), while lowering the CV-*g*_*Na*_ to levels observed in *Scn1b*^*−*/−^ neurons (Fig. 6F) resulted in spike trains that were highly stereotyped (Fig. 6G). To quantify spike train differences resulting from these two distributions, we quantified the spike train correlation, phase coherence, firing rate, and firing rate heterogeneity as in Fig. 5. The spike train correlation was 0.12 in the high variance *g*_*Na*_ group of neurons and 0.34 in the low variance *g*_*Na*_ group. The mean firing rate was 9.27 Hz and the firing rate CV was 1.12 in the high variance *g*_*Na*_ group and 8.20 Hz and 0.47, respectively in the low variance *g*_*Na*_ group. To explore the range of input fluctuations over which this effect of *g*_*Na*_ variability influences the resulting spike patterns, we varied the magnitude of input fluctuations by multiplying the σi and σe by factors between 0.5 and 3 (resulting in ranges of 0.252–1.512 nS for σi and 0.063–0.378 nS for σe, Fig. 6E). With increasing input fluctuations, both groups increased in spike correlation as in Fig. 5E, with the low variance *g*_*Na*_ group matching the high variance *g*_*Na*_ group at a 5-fold higher synaptic conductance σ. Mean population firing rates were increased by increasing input fluctuations, with similar rates in both groups across the measured range. The CV-firing rates dropped with increasing input fluctuations, with the high variance *g*_*Na*_ group reaching equal values at a 2.5-fold higher synaptic conductance σ. These results show, using a simple computational model, a strong influence of *g*_*Na*_ heterogeneity on spike synchrony and spike patterning under in vivo like fluctuations in synaptic conductances.

### *g*_*Na*_ diversity suppresses spontaneous network synchronization resulting from synaptic coupling

In the above results showing the effects of *g*_*Na*_ heterogeneity in neurons firing in response to fluctuating inputs, it was assumed that neurons receive highly correlated (in the above cases identical) inputs from an external source that is independent of the neurons targeted. In vivo, external input correlations between neurons are not identical and external inputs may vary dynamically in response to the activity of target neurons. While membrane potential fluctuations in vivo can be highly correlated, there is a broad range of input correlations that may result from different network topologies. We thus sought to examine how coupling between neurons in networks with varying topologies and synaptic weights might interact with heterogeneity of *g*_*Na*_ to influence spike correlation and patterning. A likely source of correlated membrane potential fluctuations observed in vivo is recurrent local network connectivity, where individual neurons have a high likelihood of having the same neighboring target neurons and are likely to receive reciprocal connections. Watts-Strogatz (WS) model networks provide a convenient and quantitative approach to modeling variations in such architectures, exhibiting topologies ranging from highly locally connected to random networks. Importantly, WS networks can model networks exhibiting small path lengths and high clustering, typical of small world networks observed in vivo^24,25^.

**Figure 7:**
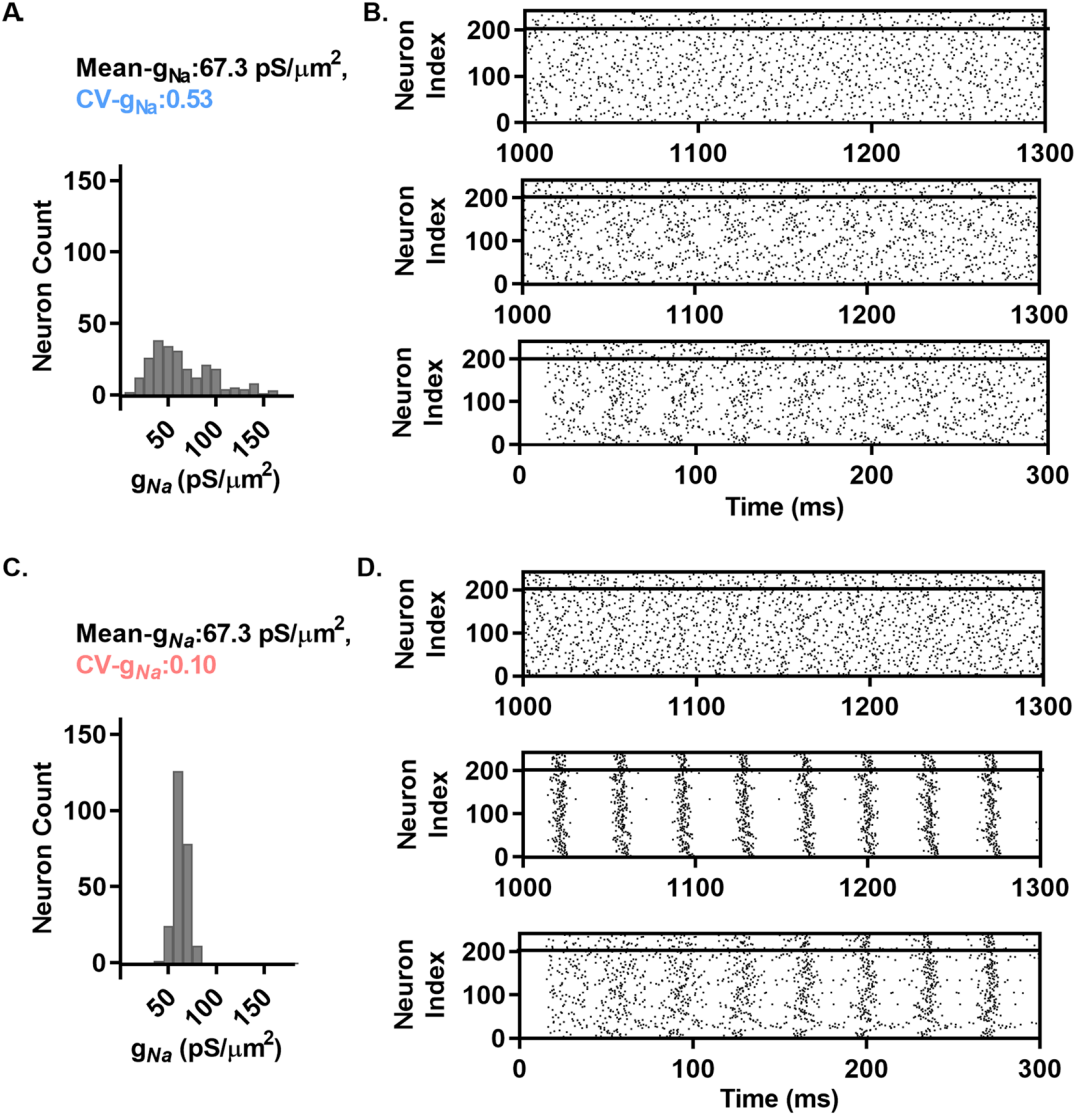
*g*_*Na*_ diversity suppresses spontaneous network synchronization of coupled neurons. (**A**) and (**C**) Histogram of the *g*_*Na*_ assigned to neurons used in the simulations shown in B. and D. CV-*g*_*Na*_ denotes the coefficient of variation in *g*_*Na*_. Distribution includes 3 values > 180 pS/µm^2^ not shown on the histogram for visibility. (**B**) and (**D**) (top graphs) Raster plots of the activity of the neurons in A. and C. with no synaptic connections between them, with firing driven by independent noise to each neuron. Middle: Raster plots of connected networks after 1000 ms with a connection radius of 6 neurons and rewiring probability of 30%. Bottom: Raster plots of activity at the beginning of the above simulations, showing synchrony onset in the CV-*g*_*Na*_ = 0.10 network but not in the CV-*g*_*Na*_ = 0.50 network. Values of F, IEI, and PC can be found in the text.

Networks were formed starting with N neurons placed in a circle, connected in a directed manner to their neighbors with radius (R) on each side. Then, with an associated probability (P), connections were rewired randomly to any other neuron in the network. We used a network with P = 30 and connection radius R = 6 for simulations throughout Fig. 7. We added 40 inhibitory neurons (5:1, excitatory:inhibitory neurons) with properties as above, with local reciprocating connections to excitatory neurons with radius = 6 neurons. Excitatory and inhibitory synaptic currents were generated as detailed in Methods.

We quantified network function using three different measures averaged over each neuron in the network, including the coefficient of variation in interevent interval (IEI-Clustering, Arb. Units) to examine divergence from randomly distributed activity of presynaptic neurons (i.e. high IEI-Clustering indicating a high clustering of presynaptic input with IEI-Clustering = 1 indicating a Poisson distribution in arrival times), the phase coherence (PC) to measure firing phase synchrony of all neurons in the network, and the mean firing rate (F, Hz). Each neuron was stimulated to sustain a background level of activity by an uncorrelated Gaussian distributed noise current injection (mean and standard deviation = 80 pA unless stated otherwise) with random (uniform distribution) time of onset over 50 ms to avoid any spurious synchrony from the beginning of simulations. In the absence of synaptic connections, this approach supports asynchronous background activity in both the high and low *g*_*Na*_ variability networks (IEI-Clustering = 0.99 ± 0.00, PC = 0.28 ± 0.00, F = 20.79 ± 0.26 Hz for CV-*g*_*Na*_ = 0.53; IEI-Clustering = 0.97 ± 0.01, Fig. 7B top and IEI-Clustering = 1.01 ± 0.00, PC = 0.46 ± 0.00, and F = 27.14 ± 0.01 Hz for CV-*g*_*Na*_ = 0.10, Fig. 7D top).

With the above conditions that support uncorrelated firing in both CV-*g*_*Na*_ conditions in the absence of coupling, we next investigated the effect of coupling neurons in small world networks as described above. To start, we connected the neurons with connection probability of 6% (radius = 6 neurons) and rewiring probability of 30%. We found that synchrony was markedly increased in the 0.10 CV-*g*_*Na*_ network (IEI-Clustering = 2.88 ± 0.10 and PC = 0.91 ± 0.02) compared to the highly desynchronized 0.53 CV-*g*_*Na*_ network (IEI-Clustering = 1.09 ± 0.04 Arb. Units, PC = 0.30 ± 0.00 Arb. Units) (Fig. 7B and D, middle). Figure 7D, bottom, shows the onset of synchrony from the random activity at the start of the simulation in the CV-*g*_*Na*_ = 0.10 network, while synchrony failed to develop in the CV-*g*_*Na*_ = 0.53 network (Fig. 7B, bottom).

**Figure 8:**
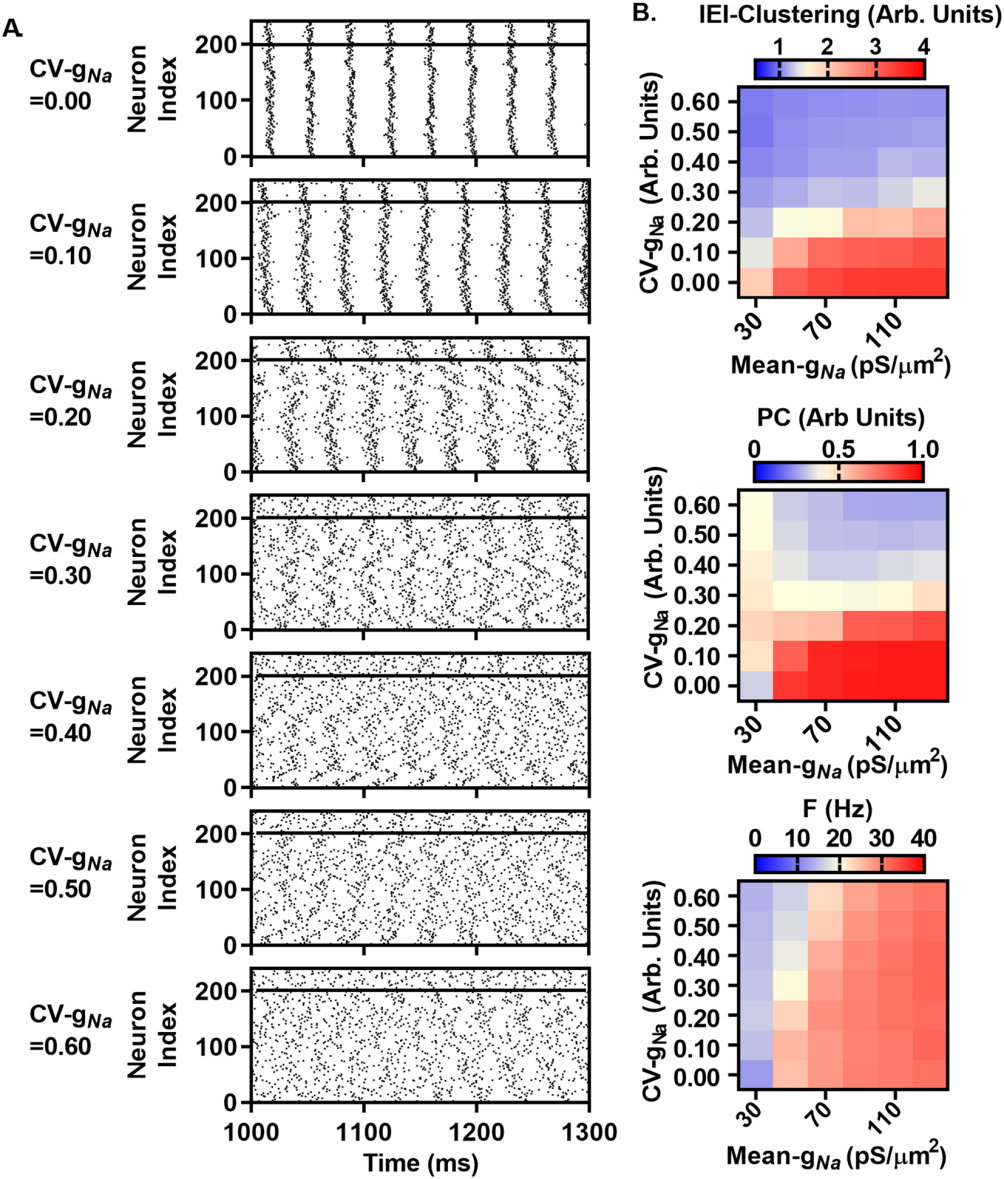
Asynchrony increases with increasing CV-*g*_*Na*_, with weak dependence on mean *g*_*Na*_. (**A**) Representative raster plots of activity in *Scn1b-*WT networks, as in Fig. 6, but with mean *g*_*Na*_ set at 70 pS/µm^2^ (from parameter sweep in B) and CV-*g*_*Na*_ increasing from top to bottom in 0.1 increments from 0 to 0.60. (**B**) Quantification of network activity as in A, with sweeps over mean *g*_*Na*_ (30–130 pS/µm^2^) and CV-*g*_*Na*_ (0–0.60), showing weak dependence of IEI-Clustering (top) and PC (middle) on mean *g*_*Na*_ but strong dependence on CV-*g*_*Na*_, while F (bottom) shows weak dependence on CV-*g*_*Na*_ and strong dependence on mean *g*_*Na*_. Note, the mean *g*_*N*a_ = 30 pS/µm^2^ group exhibited no activity when *I*_*Noise*_ = 80 pA, thus *I*_*Noise*_ was increased to 100 pA to support spontaneous firing.

We next investigated the effects of mean *g*_*Na*_ and CV-*g*_*Na*_ on network activity over a range of values (Fig. 8A and B). Representative raster plots of activity at mean *g*_*Na*_ = 70 pS/µm^2^ with CV-*g*_*Na*_ increasing in increments of 0.10 from 0.0 to 0.60 are shown in Fig. 8A, demonstrating the correspondence between increasing CV-*g*_*Na*_ and decreasing spiking synchrony, quantified in Fig. 8B. This effect occurred over various levels of mean *g*_*Na*_, quantified in Fig. 8B. Increasing mean *g*_*Na*_ resulted in increased F across different CV-*g*_*Na*_ values (Fig. 8B). Increased synchrony resulting from reduced CV-*g*_*Na*_ persisted despite the inclusion of alternative densities for *g*_*KA*_ and *g*_*KDR*_. Halving or doubling the values for *g*_*KA*_ and *g*_*KDR*_ failed to overcome the effects of CV-*g*_*Na*_ on IEI-Clustering and PC, while strongly affecting F (Table 1). We tested for the ability of CV-*g*_*Na*_ to suppress IEI-Clustering and PC with *g*_*Na*_ values sampled from either gamma or Gaussian distributions, respectively (mean = 67.3, CV-*g*_*Na*_ = 0.53 or 0.10). In the Gaussian case, resampling of values < 25 pS/um^2^ resulted in distortions to the mean *g*_*Na*_ (77.3 ± 0.60 pS/µm^2^) and CV-*g*_*Na*_ (0.38 ± 0.01). With both alternative distributions, the effect of lowered CV-*g*_*Na*_ was similar to results found using a lognormal distribution (Table 1).

**Table 1:** High CV-*g*_*Na*_ suppresses synchrony across multiple cellular conditions.

	CV-*g*_*Na*_ = 0.53	CV-*g*_*Na*_ = 0.10
*Scn1b*^*−*/−^ cell diameter		
IEI-clustering (Arb. Units)	1.14 ± 0.00	3.23 ± 0.00
PC (Arb. Units)	0.33 ± 0.00	0.92 ± 0.00
F (Hz)	31.22 ± 0.07	33.00 ± 0.02
*Scn1b*^*−*/−^ *g*_*Na*_ (I_Noise_ = 80 pA)		
IEI-clustering (Arb. Units)	0.98 ± 0.01	1.26 ± 0.01
PC (Arb. Units)	0.36 ± 0.01	0.48 ± 0.01
F (Hz)	11.67 ± 0.11	13.85 ± 0.11
*Scn1b*^*−*/−^ *g*_*Na*_ (I_Noise_ = 100 pA)		
IEI-clustering (Arb. Units)	1.06 ± 0.02	3.04 ± 0.06
PC (Arb. Units)	0.37 ± 0.00	0.92 ± 0.00
F (Hz)	22.85 ± 0.07	33.91 ± 0.08
*g*_*KDR*_ = 20 pS/µm^2^, *g*_*Na*_ = 5 pS/µm^2^		
IEI-clustering (Arb. Units)	1.09 ± 0.01	3.09 ± 0.01
PC (Arb. Units)	0.30 ± 0.00	0.93 ± 0.00
F (Hz)	28.34 ± 0.01	31.00 ± 0.03
*g*_*KDR*_ = 80 pS/µm^2^, *g*_*Na*_ = 20 pS/µm^2^		
IEI-clustering (Arb. Units)	1.09 ± 0.02	2.87 ± 0.02
PC (Arb. Units)	0.35 ± 0.01	0.88 ± 0.01
F (Hz)	22.82 ± 0.07	31.92 ± 0.02
Gamma distributed *g*_*Na*_		
IEI-clustering (Arb. Units)	1.11 ± 0.02	2.89 ± 0.02
PC (Arb. Units)	0.32 ± 0.01	0.90 ± 0.00
F (Hz)	21.89 ± 0.83	28.00 ± 0.00
Gaussian distributed g_Na_ (Resampled if *g*_*Na*_ < 25 pS/µm^2^)		
IEI-clustering (Arb. Units)	1.14 ± 0.01	2.84 ± 0.10
PC (Arb. Units)	0.34 ± 0.01	0.90 ± 0.01
F	26.10 ± 0.11	30.00 ± 0.00

Center and right columns segregate results comparing 0.53 CV-*g*_*Na*_ and 0.10 CV-*g*_*Na*_ networks (excepting specific altered condition noted in left column). Here, cell diameter is 26.2 µm in all simulations except those labeled *Scn1b*^*−*/−^, where diameter is 22.5 µm. Mean *g*_*Na*_ is 67.3 pS/µm^2^ except those labeled *Scn1b*^*−*/−^, where *g*_*Na*_ is 41.4 pS/µm2 *g*_*KDR*_ and *g*_*KA*_ are 40 and 10 pS/µm^2^, respectively, except those labeled with alternative densities where *g*_*KA*_ is fixed relative to *g*_*KDR*_ at 1:4. Gamma distribution denotes the alternative distribution used for sampling of *g*_*Na*_. Gaussian distribution denotes the alternative distribution used for sampling of *g*_*Na*_, with the added inaccuracy of required resampling of *g*_*Na*_ for low and negative values. Values were resampled if *g*_*Na*_ was < 25 pS/µm^2^, which shifts the CV-*g*_*Na*_ distribution from nominal mean 67.3 to 77.3 pS/µm^2^ and the nominal CV-*g*_*Na*_ 0.53–0.38. In all simulations, *I*_*Noise*_ was 80 pA (mean and standard deviation) unless noted, where it is increased to 100 pA.

### *g*_*Na*_ heterogeneity increases the dynamic range of network activity and shifts the network coupling requirements necessary for spontaneous network synchronization

**Figure 9:**
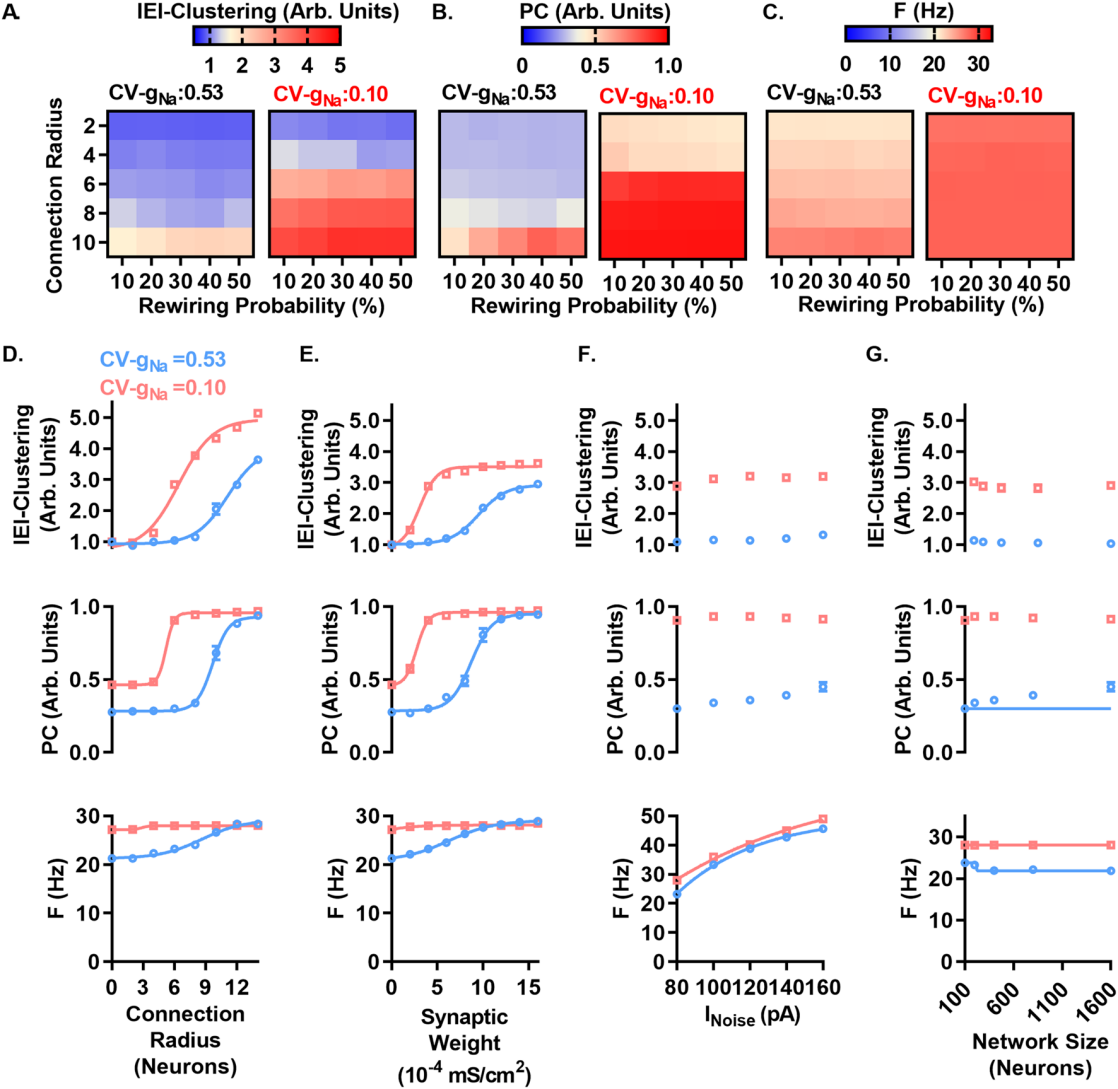
High CV-*g*_*Na*_ displaces the network coupling requirements to support synchrony and increases the dynamic range of network activity. (**A**) IEI-Clustering, (**B**) PC, and (**C**) F, across connectivity radius (R) and rewiring probability (P) in WS networks with parameters 0.53 CV-*g*_*Na*_ (left) and 0.10 CV-*g*_*Na*_ (right). Mean *g*_*Na*_ was set at (67.3 pS/µm^2^) for all simulations. Note similar IEI-Clustering between groups at low R and increasing IEI-Clustering and PC with increasing R but weak dependence on P. F (right) increases with increasing R in the CV-*g*_*Na*_ = 0.53 case while F remains similar across all R in the 0.10 CV-*g*_*Na*_ cases, showing insensitivity of low CV-*g*_*Na*_ networks to respond to changing network conditions. (**D**) Sweep over R with P fixed at 30% from 0 to 14 neurons showing high CV-*g*_*Na*_ displaces the dependence of IEI-Clustering (top) and PC (middle) on R to higher levels. F (bottom) shows high CV-*g*_*Na*_ networks change F with increasing R, while low CV-*g*_*Na*_ networks maintain similar F across R. Line represents fit with Boltzmann equation throughout figure. (**E**) Dependence of IEI-Clustering (top), PC (middle), and F (bottom) as in D, but sweeping over weight of synaptic connections from 0.0000 to 0.0016 mS/cm^2^ while R is fixed at 6, showing similar displacement of IEI-Clustering (top) and PC (middle) as in D. F (bottom) shows similar insensitivity to changing network conditions in 0.10 CV-*g*_*Na*_ networks while 0.53 CV-*g*_*Na*_ networks increase frequency with increasing synaptic weight. (**F**) Dependence of IEI-Clustering and PC on *I*_*Noise*_, sweeping (mean and standard deviation) from 80 to 160 pA. In contrast to altering internal network conditions as in D and E, increasing the amount of external stimulation does not strongly affect IEI-Clustering (top) and PC (middle) while F (bottom) in both 0.53 and 0.10 CV-*g*_*Na*_ groups is strongly affected, suggesting that the alterations shown in D and E are effects of network coupling, rather than increased excitation. (**G**) Dependence of IEI-Clustering (top), PC (middle) and F (bottom) on network size with fixed P (30%) and R (6 neurons) sweeping from 100 to 1600 neurons, showing relative insensitivity to network size.

We next tested the capacity of CV-*g*_*Na*_ to suppress network synchrony across different network conditions by comparing a network with mean *g*_*Na*_ = 67.3 pS/µm^2^ and CV-*g*_*Na*_ = 0.53 to the same network with CV-*g*_*Na*_ = 0.10. Across all simulations with various P and R, the 0.1 CV-*g*_*Na*_ network exhibited higher IEI-Clustering and PC (Fig. 9A and B). At low R however, the 0.1 CV-*g*_*Na*_ networks approached IEI-Clustering and PC levels similar to CV-*g*_*Na*_ = 0.53 network. While the 0.53 CV-*g*_*Na*_ networks exhibited increasing F with increasing R (up to 27% increase), the 0.10 CV-*g*_*Na*_ networks exhibited only minor changes in F with increasing R (up to a 3% increase) (Fig. 9C). To quantify the relative network requirements to support synchrony under high and low CV-*g*_*Na*_ conditions, we performed simulations with increasing R and with P fixed at 30%. The 0.53 CV-*g*_*Na*_ networks required R to be increased to 12 to reach levels of IEI-Clustering and PC found in the 0.10 CV-*g*_*Na*_ network model at R = 6 (i.e. a 2 fold increase) (Fig. 9D). Similarly, varying the synaptic weights showed that the 0.53 CV-*g*_*Na*_ networks required synaptic weights to be increased by ~ 2 fold to approach the IEI-Clustering and PC values observed in the 0.10 CV-*g*_*Na*_ network (Fig. 9E). F was again relatively insensitive to increasing R and synaptic weights in the 0.10 CV-*g*_*Na*_ networks but increased with increasing R or synaptic weights in the 0.53 CV-*g*_*Na*_ networks, converging with the 0.1 CV-*g*_*Na*_ networks at higher values (Fig. 9E). To examine the dependence of high CV-*g*_*Na*_ suppression of network synchrony on specific firing rates, we tested various amplitudes of external drive. We found that increasing *I*_*Noise*_ had minor effects on IEI-Clustering and PC but resulted in large increases in F (Fig. 9F). *I*_*Noise*_ below 80 pA failed to support any spiking in the 0.10 CV-*g*_*Na*_ network and supported only a small number of neurons in the 0.53 CV-*g*_*Na*_ network. We performed simulations sweeping across network size with P = 30 and R = 6 and found that IEI-Clustering and PC remained higher in 0.10 CV-*g*_*Na*_ networks compared to 0.53 CV-*g*_*Na*_ networks at all network sizes tested (Fig. 9G).

**Figure 10:**
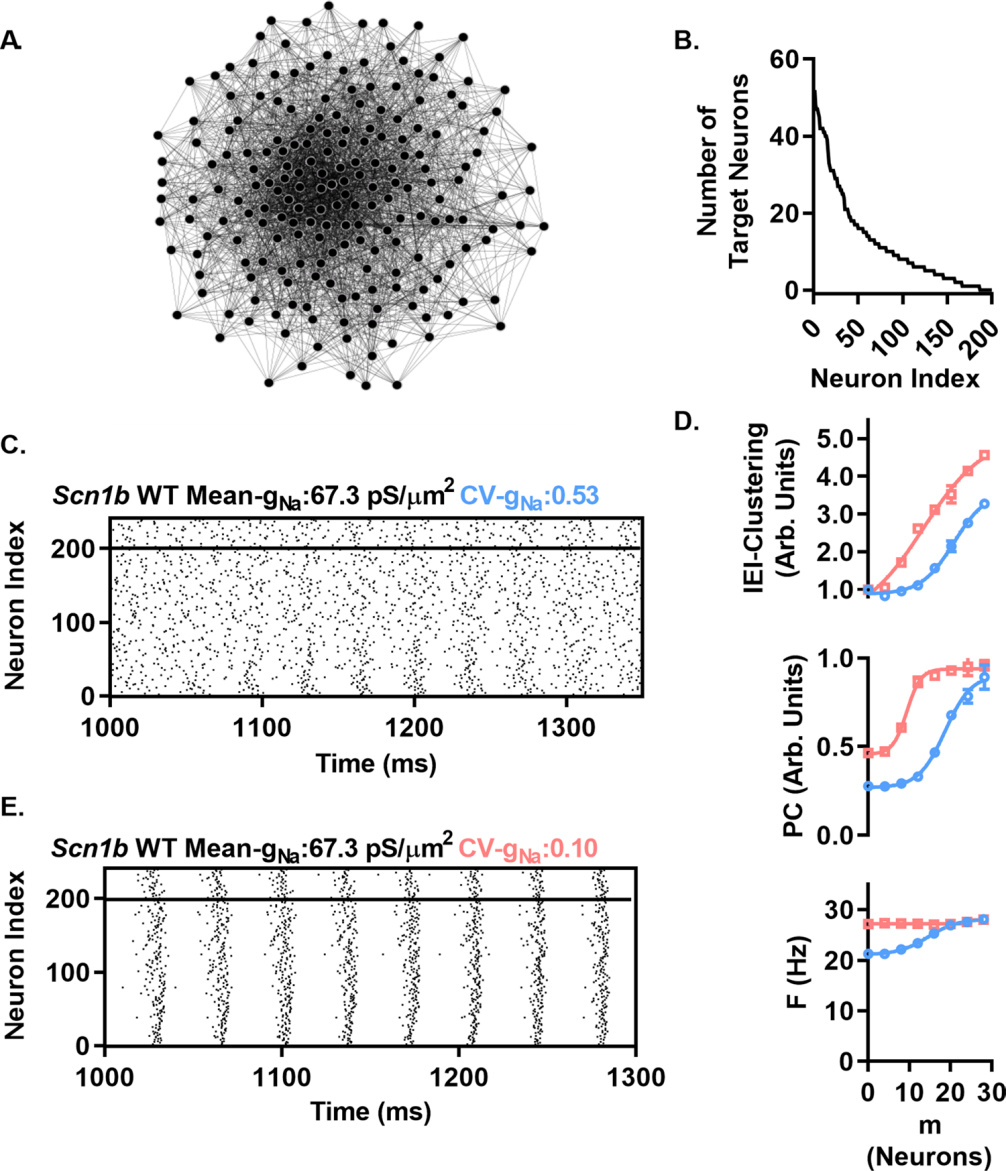
High CV-*g*_*Na*_ displaces the network coupling requirements to support synchrony in networks with heterogeneous connection probability. (**A**) Graphical representation of a representative Scale-Free like network structure of N = 200 excitatory neurons. Visualization generated in Gephi v0.9.2 https://github.com/gephi/gephi/releases. (**B**) Histogram of the number of target excitatory neurons for each excitatory neuron in the network shown in A, exhibiting the heavy tailed distribution characterizing scale free networks. (**C**) Representative raster plots of m = 12 networks with CV-*g*_*Na*_ = 0.53 (top) or 0.10 (bottom) and mean 67.3 pS/µm^2^. Note in 0.53 CV-*g*_*Na*_ network, transient synchrony observable in neurons with low index (high out-degree neurons) which grows over ~ 100 ms but is dissipated shortly after reaching peak while the 0.10 CV-*g*_*Na*_ network maintains network wide synchrony. (**D**) IEI-Clustering (Top), PC (Middle) and F (Bottom) resulting from sweeping from m = 0 to 30 neurons, showing similar to WS networks investigated in 3.6 CV-*g*_*Na*_ = 0.10 networks have lower network requirements (here m) required to support neuronal synchrony. Again, similar to WS networks, F in the 0.53 CV-*g*_*Na*_ networks is sensitive to altering network connectivity while the F in the 0.10 CV-*g*_*Na*_ networks is insensitive.

The simulations above model several different network types by varying R between high and low values including regular, small-world, and random networks. While in vivo neuronal networks exhibit small world like characteristics like those investigated above, there is also evidence that they exhibit heterogeneous distributions in the number of synaptic connections. In particular, there is evidence of power-law or “scale-free” like distributions in connection probability, i.e. there exist neuronal “hubs” with a low number of highly connected neurons and a high number of neurons with much fewer connections^26^. Thus, we asked whether CV-*g*_*Na*_ also impacts the network requirements for synchronization in networks with these characteristics. We generated directed scale-free like networks as detailed in^27^. Briefly, directed networks of size N with power-law like distribution in synaptic connection probability were generated by starting with a seed network of m neurons with all-to-all connectivity. Then iteratively, N-m neurons were added to the network with each new neuron j receiving m connections from m different pre-existing k_i_ neurons in the network. Selection of neurons for k_i_ was random, with probability proportional to the existing number of target neurons post-synaptic to neuron k_i_. This resulted in a heterogeneous connection probability, with few neurons having a high connection probability and a greater number with fewer connections. A graphical representation of a network with N = 200 and m = 12 is shown in Fig. 10A. The accompanying distribution in out-degree is shown in Fig. 10B. 40 inhibitory neurons were added as above for WS networks, with local (from neuron index) connection radius of 6 neurons. Representative raster plots for the 0.53 CV-*g*_*Na*_ and 0.1 CV-*g*_*Na*_ networks are shown in Fig. 10C and E respectively. To examine the relative requirements for synchronization in 0.10 and 0.53 CV-*g*_*Na*_ networks, we swept across m from 0 to 28 neurons. Similar to our findings for WS networks, we found that 0.10 CV-*g*_*Na*_ networks exhibit higher IEI-Clustering and PC across a variety of network conditions, with 0.53 CV-*g*_*Na*_ networks requiring ~ 2 fold higher m to achieve similar degrees of IEI-Clustering and PC as the 0.10 CV-*g*_*Na*_ network (Fig. 10D, top and middle). Similar to the WS networks, firing rate in the 0.1 CV-*g*_*Na*_ network remained relatively unaffected by changing network connectivity, while in the 0.53 CV-*g*_*Na*_ network, F increased with increasing m (Fig. 10D, bottom). These results show that high *g*_*Na*_ diversity raises the network connectivity requirements to support neuronal synchrony in networks with heterogeneous degree distribution.

## Discussion

Here, we use genetic, pharmacological, and computational approaches to show that variability in *I*_*Na*_ density between pyramidal neurons contributes to low spike correlation, variability in stimulus feature selection, and suppression of network synchronization. Taken together, our work provides new insights on the importance of *I*_*Na*_ density heterogeneity in neural coding and network synchronization, as well as contexts for the impact of impaired ion channel heterogeneity on network function. While variability in ion channel density between neurons is well-established in a variety of systems, whether this variability is a target state for the brain, or the result of biological imprecision, is not known. Similarly, the large amount of noise and low correlation in neuronal firing between neurons tuned to the same stimulus suggests that spike timing precision between neurons is not a priority under normal brain function^28^. This idea is often at odds with our understanding of spike timing precision and neuronal encoding, and is a particular issue for VGSCs where large differences in VGSC properties may result in delays of spike initiation in the millisecond range, well below the apparent level of precision at which the brain operates in vivo^29^. The observation that neurons are capable of high spike timing precision in response to noisy current injections established that neurons are at least equipped to generate this high level of precision^30^. Why do correlated membrane potential fluctuations not then result in highly correlated spike times in vivo? One possible contributor for this low correlation is biophysical heterogeneity^11^. Our work shows that normal variations in *I*_*Na*_ density between neurons have a large impact on spike patterning in response to a noisy stimulus, thus expanding the role of VGSCs in neuronal coding. This variability contributes to the widely observed decorrelated state of neuronal firing in the healthy brain. Furthermore, our work shows that *I*_*Na*_ density variability between neurons in the mammalian brain is dependent upon *Scn1b*, encoding VGSC β1/β1B subunits, demonstrating that this decorrelated state is actively supported by specific ion channel regulatory mechanisms rather than a result of biological disorganization. Finally, our observations of impaired variability between neurons and impaired decorrelation occurring spontaneously in an animal model of disease suggest that decorrelated firing and the processes that support it are crucial to maintaining normal brain function.

### Ion channel variability

In contrast to genes encoding other voltage-gated channels, the repertoire of VGSC genes expressed in the brain is relatively limited and VGSCs do not vary as dramatically in their voltage dependent or kinetic properties, e.g.^31^. We have focused here on spiking mechanisms that can be considered fluctuation driven, i.e. irregular spiking in response to fluctuating inputs^32^. While the repertoire of voltage-gated channels in brain is extensive, suggesting a high combinatorial capacity to promote spike pattern diversity, all such channels in the mammalian brain must ultimately interact with VGSCs to generate an action potential. Thus, VGSCs are the final step in translating membrane potential fluctuations into spike outputs. We show here, using genetic, pharmacological, and computational models, that spiking in response to fluctuating stimuli is dependent on modest changes in *I*_*Na*_ density, which varies over a wide range between neurons, resulting in decreased firing correlation for the same inputs. Future work may reveal specific roles of individual VGSC subtypes, or variability in combination with other ion channels, which are also expected contribute to this spiking mechanism. Additionally, there are likely compensatory mechanisms that balance spike pattern variability, e.g. total firing rate and synaptic input constraints. Future work may reveal how variability at the network level and in other voltage dependent conductances may impact spike pattern generation. Notably, at the single neuron level, spiking features that are dependent upon multiple conductances have restrictions that can be compensated by different families of voltage gated channels^33^. Importantly, despite the potential roles of these other conductances, we demonstrate a functional consequence of *I*_*Na*_ variability loss in the decorrelation of firing in multiple pyramidal neuron populations, as well as in juvenile and adult mice, when the *I*_*Na*_ variability-enhancing VGSC gene *Scn1b* is deleted.

### Cell extrinsic vs. cell intrinsic impacts on spike train decorrelation

The possibility of diverse dendritic morphologies and synapse locations limiting input correlations are important to consider. However, in vivo paired whole cell recordings have shown that neighboring neurons exhibit high membrane potential correlation^13,14^. This previous work showed that, even though no two neurons have identical dendritic morphology, location, or synaptic strength, synaptic input in vivo is capable of converging to similar fluctuations in membrane potential within neighboring neurons. Despite these high membrane potential correlations, spiking is largely asynchronous in L2/3 pyramidal neurons^34^ and in the cortex in general^16^. It follows that neuronal firing in response to similar membrane potential fluctuations may in large part be due to intrinsic biophysical differences such as those described here for *I*_*Na*_ density. We propose that *I*_*Na*_ density heterogeneity between neurons is a crucial contributor in converting similar input fluctuations into dissimilar spike outputs. Furthermore, we show that *g*_*Na*_ variability between model neurons actively suppresses spiking synchronization that is generated through network coupling by increasing the strength of connections or network topology.

### Cell type specificity of heterogeneity

We showed here and previously that the level of *I*_*Na*_ density within individual populations of wildtype neurons is variable. A major current focus in neuroscience is to identify and understand the properties of specific neuronal cell types. These efforts have resulted in classifying multiple subtypes of inhibitory interneurons and laminar differences between pyramidal neurons and/or their targets. One interpretation of our results may be that there exist discrete subclasses of pyramidal neurons with tightly controlled levels of *I*_*Na*_ density within cortical layers and hippocampal regions and that certain populations are lost or fail to develop properly in *Scn1b*^*−*/−^ mice. However, previous work has shown similar ranges of *I*_*Na*_ density heterogeneity in nucleated patches from L5 and L6 pyramidal neurons, GABAergic and dopaminergic neurons of the substantia nigra, and as shown here, subicular pyramidal neurons^8–10^. The functional importance of further subclass distinction among pyramidal neurons in *I*_*Na*_ density is unclear when this similar variation recurs over different cortical layers, brain regions, and neurotransmitter type. It is possible that discrete cell types within cortical layers and hippocampal regions respond differently to fluctuations utilizing specific *I*_*Na*_ densities to direct the response of neuronal spiking based on their downstream targets and/or specialized functions. There may be precedence for such ion channel regulation in the level of expression of *I*_*h*_ in different glomeruli of the olfactory bulb^35^. Conversely, even in model organisms where the precise identity of individual neurons is unambiguous, the expression levels of specific ion channels vary between different animals, which can be combined across different conductances to achieve similar functional outputs^6,36,37^.

### Epilepsy, hyperexcitability, and hypersynchrony

A future goal of our work is to understand the consequences of variable *I*_*Na*_ density between neurons in terms of neuronal synchrony vs. hyperexcitability in seizure mechanisms. *Scn1b*^*−*/−^ mice, which model early infantile developmental and epileptic encephalopathy (OMIM 617350, DEE52) demonstrate the powerful influence of *I*_*Na*_ density variability on spiking correlation and neuronal synchronization. While the effects of altered VGSC expression in epilepsy models have often been interpreted within the context of excitatory/inhibitory imbalance, our observation of an additional role in spike patterning and neuronal synchrony expands this discussion to consider other roles that VGSCs may play in shaping network activity. Interestingly, studies in human epileptic tissue have found evidence of decreased functional heterogeneity in rheobase, which was modeled computationally to demonstrate an impaired resilience to neuronal synchronization^38^. Thus, neuronal *I*_*Na*_ density heterogeneity and its role in spike decorrelation may be a crucial mediator of these deficits.

### Network structure and *I*_*Na*_ variability

In conclusion, our work shows that variable levels of *g*_*Na*_ between neurons displaces the network coupling requirements to support synchronization across a range of network topologies and synaptic strengths. Importantly, we show that varying network topology and connection strengths confers a broader range of network activity patterns in heterogenous *g*_*Na*_ networks than in homogenous *g*_*Na*_ networks. Driving neurons to fire more with increased exogenous drive did not significantly interact with *g*_*Na*_ variability to affect firing frequency, phase coherence, or presynaptic activity clustering. However, stronger coupling strength and varying network topology did have robust influences in the patterning of network activity in a *g*_*Na*_ variation dependent manner. Our results suggest that *g*_*Na*_ variability acts in concert with network structure to shape network activity patterns, an effect that may be dynamically controlled through the influence of neurotransmitter systems, drugs, genetic lineage including the expression of gene variants or modifiers, or other mechanisms that control the availability of VGSCs.

## Methods

### Animals

All experiments were performed in accordance with NIH and ARRIVE guidelines and approved by the University of Michigan Institutional Animal Care and Use Committee. *Scn1b*^+/+^ and *Scn1b*^*−*/−^ mice were generated as described^39^ and were congenic on the C57BL/6J background for over 20 N generations. *Scn1b*^*Fl/Fl*^ mice, on the C57BL/6J background, were generated as described^40^ and crossed with *Slick-H* mice (JAX Tg(Thy1-cre/ERT2,-EYFP)HGfng/PyngJ, stock #012708) received on the C57BL/6J × CBA F1 background and then backcrossed to C57BL/6J, to generate *Slick-H*/*Scn1b*^Fl/Fl^ mice. Tamoxifen (TMX) was purchased from Sigma-Aldrich and dissolved in sunflower oil + 2% ethanol at a final concentration of 10 µg/µl*.* 10 µl per g of mouse weight of TMX or vehicle was administered IP once per day for 4 consecutive days.

### Brain slice preparation

Acute brain slices were prepared as described^9^. Mice were anesthetized with isoflurane, decapitated, and brains were removed and placed in 95:5% O_2_:CO_2_ continuously aerated ice-cold slice solution containing in mM: (110 sucrose; 62.5 NaCl; 2.5 KCl; 6 MgCl_2_; 1.25 KH_2_PO_4_; 26 NaHCO_3_; 0.5 CaCl_2_; and 20 D-glucose (pH 7.35–7.40 when aerated at RT). 300 µm thick coronal sections were cut from the visual cortex region. Slices were incubated in an aerated holding chamber containing slice solution for 30 min at 34 °C and then incubated in a mixture of artificial cerebrospinal fluid (ACSF) and slice solution (1:1) for 30 min at room temperature. ACSF contained in mM (125 NaCl; 2.5 KCl; 1 MgCl_2_; 1.25 KH_2_PO_4_; 26 NaHCO_3_; 2 CaCl_2_; and 20 D-glucose (pH 7.35–7.40 with aeration). Slices were then aerated with 100% ACSF for at least 30 min before use.

### Action potential recording and analysis

During recording, slices were superfused with 2–3 ml/min aerated ACSF at 34 °C. Neurons were visualized using a Nikon A1R upright confocal microscope equipped with IR-DIC optics with a 40X water immersion objective. Only vertically oriented pyramidal cells were selected for recording. Recording electrodes had a resistance of 3–6 MΩ with solutions containing in mM (140 K-Gluconate, 4 NaCl, 0.5 CaCl_2_, 10 HEPES, 5 EGTA, 5 phosphocreatine, 2 Mg-ATP, and 0.4 GTP (pH adjusted to 7.2–7.3 with KOH). The junction potential was calculated to be 14.3 mV using the P-clamp junction potential calculator in the whole cell mode and all values were corrected offline, with all values presented in the study as corrected values. The resting membrane potential was defined as the membrane potential in current clamp less than 10 s after initial break in. Data were acquired at 20 kHz and were filtered at 10 kHz. Cells with an access resistance measured in voltage clamp greater than 20 MΩ or RMP greater than − 64.3 mV were discarded. Access resistance and pipette capacitance were compensated using bridge balance. Action potentials were defined as the voltage crossing − 20 mV subsequent to a dv/dt > 10 mV/ms. Input resistance was calculated using Ohm’s law with 10 pA current injection from the resting membrane potential after 250 ms. Cells that had changes in access resistance greater than 20% of the value between the start and end of recordings were discarded.

### Nucleated patch clamp recording

Nucleated patches were pulled as described previously^9^. Recordings were performed at 23 °C.

### Perforated patch clamp

Perforated patch clamp was performed in the same recoding conditions as whole cell recordings above. Amphotericin B stock solution was prepared by dissolving in DMSO at a concentration of 50 mg/ml and sonicating. Stock solution was then added to the whole cell recording internal solution above to give a final concentration of 100 µg/ml and sonicating. Recording pipettes tips were then filled with recoding solution above and pipettes were backfilled with solution Amphotericin B containing internal solution. After the establishment of a GΩ seal, cells were allowed to perforate for between 15 and 30 min until access resistance less than 30 MΩ was stable (less than 10% change) for 5 min. Input resistance was monitored by a − 10 pA hyperpolarizing current injection between sweeps and cells were discarded if input resistance changed by more than 20% during the duration of the recording. Cells with access resistance changes greater than 20% throughout the recording were similarly discarded.

### Statistics

No more than 1 cell was acquired per slice, no more than 4 cells were acquired per animal, and each experiment was performed with at least 4 animals. Each cell is reported as n = 1 and each animal is reported as N = 1. For measures of spike train correlation or phase coherence between trials within the same cell, the pairwise cross correlation and phase coherence was measured between all trials and averaged to generate a single value (phase coherence or correlation) for each cell (n = 1). Then between groups comparisons (e.g. *Scn1b*^+/+^ vs *Scn1b*^*−*/−^) were calculated by comparing the average phase coherence or average correlation values between groups. For measures of between cell correlation or phase coherence, pairwise comparisons between trials of cell A were compared to all trials of cell B, C, etc. and the average was taken to generate a single value of phase coherence and correlation for cell A. Then between groups comparisons (e.g. *Scn1b*^+/+^ vs *Scn1b*^*−*/−^) were calculated by comparing the average phase coherence or correlation between groups. Significance was set at *p* < 0.05. Comparisons were made with an unpaired two-tailed Student’s t-test or a Mann–Whitney test, as noted in figure legends. Comparisons with application of TTX were made using a paired t-test. Outlier detection was performed with a two tailed Grubbs test with significance set at *p* < 0.01. All listed p-values are tested for significance after multiple comparisons and reported as non-significant if q > 0.05 using the Benjamini, Krieger, and Yekutieli two stage step-up method with a 5% false discovery rate correction. Values were tested for a non-Gaussian distribution using D’Agostino-Pearson omnibus normality test. Differences in variance were tested using an F-Test.

### Computational modeling

All simulations were performed in the NEURON, version 7.4, simulation environment. The model equations were based on a previous model used in the investigation of the interplay between heterogeneous intrinsic neuronal properties and network structure. We modified this model to take into account (but not to explicitly model) adolescent layer 6 pyramidal neurons *g*_*Na*_, input resistance and resting membrane potential, and to examine the impact of *g*_*Na*_ diversity in the relative context of *Scn1b*^+/+^ and *Scn1b*^*−*/−^ differences. Simulations were performed using the following current balance equation:

1 $$C_{m} \frac{{dV_{m} }}{dt} = - g_{Na} m^{3} h\left( {V_{m} - E_{Na} } \right) - g_{{K_{DR} }} n^{4} \left( {V_{m} - E_{K} } \right) - g_{{K_{A} }} qr\left( {V_{m} - E_{K} } \right) - g_{L} \left( {V_{m} - E_{leak} } \right) - I_{Syn} + I_{Noise}$$

where V_m_ is the membrane voltage; C_m_ is the cell capacitance calculated using specific capacitance of 0.9 µF/cm^2^ and surface area from morphologies as detailed below; *g*_*x*_ are the maximum conductances of the respective ionic currents; *E*_*x*_ are the equilibrium potentials for respective ions; and m, h, n, q, and r are gating functions detailed below. *E*_*Na*_ was set at 75 mV, *E*_*k*_, − 90 mV, and *E*_*leak*_ at − 81.7 or − 76.8 mV for *Scn1b*^+/+^ or *Scn1b*^*−*/−^ simulations, respectively. Gating functions followed the general form

2 $$\frac{dx}{{dt}} = \frac{{x_{inf} \left( {V_{m} } \right) - x}}{{\tau_{x} \left( {V_{m} } \right)}}$$

where *x*_*inf*_ and *τ*_*x*_ are given by gating variables detailed below. Here, F is Faraday’s constant, R is the gas constant, and T is temperature in K. Simulations were performed at a nominal temperature of 37 °C with rates defined at 23 °C with temperature dependent rates adjusted according to q10 = 2.3 (*I*_*Na*_), 5 (*I*_*KA*_), and 1 (*I*_*KDR*_). Note voltage dependent rates in *I*_*Na*_ were adjusted as in source model with an input voltage shift of − 10 mV^8^. For I_Na_, values were adjusted such that the *h*_*inf*_ curves in a 10 µm diameter and length model cell at 23 °C were within 0.1 of their respective units of the slope factor and V_1/2_ recorded in nucleated patches reported previously.

*I*_*Na*_ was calculated according to:

3 $$I_{Na} = g_{Na} m^{3} h\left( {V_{m} - E_{Na} } \right)$$

With steady state activation function:

4 $$m_{inf} \left( {V_{m} } \right) = \frac{{\alpha_{m} \left( {V_{m} } \right)}}{{\alpha_{m} \left( {V_{m} } \right) + \beta_{m} \left( {V_{m} } \right)}}$$

and activation time constant:

5 $$\tau_{m} \left( {V_{m} } \right) = \frac{1}{{\alpha_{m} + \beta_{m} }}$$

6 $$\alpha_{m} \left( {V_{m} } \right) = 0.182\left( {V_{m} + 36.42} \right)/\left( {1 - e^{{ - \frac{{V_{m} + 36.42}}{6.8}}} } \right)$$

7 $$\beta_{m} \left( {V_{m} } \right) = 0.124\left( {V_{m} + 36.42} \right)/\left( {e^{{\frac{{V_{m} + 36.42}}{6.8}}} - 1} \right)$$

With Steady State Inactivation Function:

8 $$h_{inf} \left( {V_{m} } \right) = \frac{1}{{1 + e^{{\begin{array}{*{20}c} {(V_{m} + 65.7)} \\ {/5.25 } \\ \end{array} }} }}$$

where

9 $$\alpha_{h} \left( {V_{m} } \right) = \frac{{0.024\left( {V_{m} + 50} \right)}}{{1 - e^{{ - \frac{{V_{m} + 50}}{5}}} }}$$

10 $$\beta_{h} \left( {V_{m} } \right) = 0.0091\left( {V_{m} + 75} \right)/\left( {e^{{\frac{{V_{m} + 75}}{5}}} - 1} \right)$$

Inactivation time constant function:

11 $$\tau_{h} \left( {V_{m} } \right) = \frac{1}{{\alpha_{h} \left( {V_{m} } \right) + \beta_{h} \left( {V_{m} } \right)}}$$

Delayed rectifier type K^+^ current was calculated according to:

12 $$I_{{K_{DR} }} = g_{{K_{DR} }} n^{4} \left( {V_{m} - E_{K} } \right)$$

With steady state activation function:

13 $$n_{inf} \left( {V_{m} } \right) = \frac{1}{{1 + \gamma_{n} \left( {V_{m} } \right)}}$$

And activation time constant function:

14 $$\tau_{n} \left( {V_{m} } \right) = {\text{max}}\left[ {\frac{{50\delta_{n} \left( {V_{m} } \right)}}{{1 + \gamma_{n} \left( {V_{m} } \right)}},2} \right]$$

where

15 $$\gamma_{n} \left( {V_{m} } \right) = e^{{10^{ - 3} * - 3*\left( {V_{m} - \left( { - 10} \right)*\frac{F}{RT}} \right)}}$$

16 $$\delta_{n} \left( {V_{j} } \right) = e^{{10^{ - 3} * - 2.1*\left( {V_{m} - \left( { - 10} \right)*\frac{F}{RT}} \right)}}$$

A-type K^+^ current was generated according to:

17 $$I_{{K_{A} }} = g_{{K_{A} }} qr\left( {V_{m} - E_{K} } \right)$$

with steady state activation function:

18 $$q_{inf} \left( {V_{m} } \right) = \frac{1}{{1 + \gamma_{q} \left( {V_{m} } \right)}}$$

and activation time constant function:

19 $$\tau_{q} \left( {V_{m} } \right) = {\text{max}}\left[ {\frac{{4\delta_{q} \left( {V_{m} } \right)}}{{1 + \gamma_{q} \left( {V_{m} } \right)}},0.1} \right]$$

where

20 $$\gamma_{q} \left( {V_{m} } \right) = e^{{10^{ - 3} ( - 1.5 - \left( {1 + e^{{\left( {V_{m} + 40} \right)/5}} } \right))^{ - 1} \left( {V_{m} - 11} \right)\frac{F}{RT}}}$$

21 $$\delta_{q} \left( {V_{m} } \right) = e^{{10^{ - 3} *0.55( - 1.5 - \left( {1 + e^{{\left( {V_{m} + 40} \right)/5}} } \right))^{ - 1} \left( {V_{m} - 11} \right)\frac{F}{RT}}}$$

Steady state inactivation function:

22 $$r_{inf} \left( {V_{m} } \right) = \frac{1}{{1 + \gamma_{r} \left( {V_{m} } \right)}}$$

and time constant function:

23 $$\tau_{r} \left( {V_{m} } \right) = \max \left[ {0.26\left( {V_{m} + 50} \right),2} \right]$$

where

24 $$\gamma_{r} \left( {V_{m} } \right) = e^{{10^{ - 3} *3\left( {V_{m} + 56} \right)\frac{F}{RT}}}$$

*g*_*Na*_ was set at the experimentally measured conductance densities in nucleated patches at − 20 mV, corrected by 14% for incomplete activation at − 20 mV (67.3 pS/µm^2^ for *Scn1b*^+*/*+^ and 41.4 pS/µm^2^ for *Scn1b*^*−*/−^) as calculated from nucleated patches recorded previously^9^. Note conductance is used in the model for simplicity and continuity with the source models while permeability is used experimentally for the measurement of steady state activation due to prominent rectification at higher test potentials. Comparison of the experimental mean *Scn1b*^+/+^ conductance density to values recorded in nucleated patches previously in layer 5 pyramidal neurons shows a similar density where the 50% open channel probability corrected density was 60 pS/µm^2^ (here 67.3 pS/µm^2^) and a similar range of 27–136 pS/µm^2^ (here 32.7–130.2 pS/µm^2^)^8^.

Cell morphology consisted of a single isopotential cylindrical compartment with equal diameter and length. Cell diameter was determined such that C_m_ was equal to whole cell capacitance measured experimentally for layer 6 pyramidal neurons previously in *Scn1b*^+/+^ (diameter = 26.2 µm yielding a capacitance of 19.5 pF) or *Scn1b*^*−*/−^ (diameter = 22.5 µm yielding a capacitance of 14.3 pF). R_m_ (1/g_L_) was set at 10.1 kΩ cm^2^ such that the input resistance calculated by a − 10 pA current injection at resting membrane potential in the *Scn1b*^+/+^ neuron was equal to 460 MΩ (experimental IR = 462 MΩ), yielding an *Scn1b*^*−*/−^ neuron input resistance of 627 MΩ (experimental IR = 669 MΩ). The ratio of *g*_*KDR*_ to *g*_*KA*_ was fixed in all simulations at 4. The density of *g*_*KDR*_ was determined by sweeping from 10 pS/µm^2^ to a max of 200 pS/µm^2^, setting the density at 40 pS/µm^2^ for further simulations, as this value results in depolarization block in *Scn1b*^+*/*+^ and *Scn1b*^*−*/−^ models at similar current injection ranges recorded experimentally. Doubling or halving this density did not significantly change the effect of high or low CV-*g*_*Na*_ on network synchrony but altered F, with the *g*_*KDR*_ = 80 pS/µm^2^ group requiring increased external stimulation to maintain network activity (Table 1).

Synaptic currents were generated as in^21^. When a presynaptic cell V_m_ crossed 0 mV an exponentially decaying synaptic current in the target cell was generated according to:

25 $$I_{syn} = wexp\left( { - \frac{{\left( {t - t_{i} } \right)}}{\tau }} \right)\left( {V_{m} - E_{syn} } \right)$$

where *E*_*syn*_ = 0 or − 80 mV for excitatory and inhibitory synapses respectively, τ = 0.5 or 1.5 ms for excitatory or inhibitory synapses respectively, t_i_ is the presynaptic spike time, and *w* is the synaptic weight set at a default 0.0004 mS/cm^2^, selected such that excitatory synapses provide a ~ 2 mV depolarization at the resting membrane potential in a *Scn1b*^+/+^ style model neuron. The effect of different synaptic weights on network function are investigated in Fig. 9E. Neuronal activity was supported by a background Gaussian distributed current injection *I*_*Noise*_, generated independently for each neuron with a new value sampled at each time step (0.0125 ms). Time of onset was generated randomly (from a uniform distribution) over 50 ms at the start of network simulations for each neuron to avoid synchronous activation at simulation initialization. With the exception of simulations noted in text and figure legends, all simulations used a noise with an equal mean and standard deviation of 80 pA (the minimum required in the majority of simulations to maintain network activity), with the above special cases increased to 100 pA to maintain network activity.

## Data Availability

The datasets generated and/or analyzed during the current study are available from the corresponding author on reasonable request.
